# A new framework for MR diffusion tensor distribution

**DOI:** 10.1038/s41598-021-81264-x

**Published:** 2021-02-02

**Authors:** Kulam Najmudeen Magdoom, Sinisa Pajevic, Gasbarra Dario, Peter J. Basser

**Affiliations:** 1 Division on Translational Imaging and Genomic Integrity, Eunice Kennedy Shriver, National Institute of Child Health and Human Development, National Institutes of Health, Bethesda, MD USA; 2 Department of Mathematics and Statistics, University of Helsinki, Helsinki, Finland

**Keywords:** Imaging, Nervous system, Computational science

## Abstract

The ability to characterize heterogeneous and anisotropic water diffusion processes within macroscopic MRI voxels non-invasively and in vivo is a desideratum in biology, neuroscience, and medicine. While an MRI voxel may contain approximately a microliter of tissue, our goal is to examine intravoxel diffusion processes on the order of picoliters. Here we propose a new theoretical framework and efficient experimental design to describe and measure such intravoxel structural heterogeneity and anisotropy. We assume that a constrained normal tensor-variate distribution (CNTVD) describes the variability of positive definite diffusion tensors within a voxel which extends its applicability to a wide range of b-values while preserving the richness of diffusion tensor distribution (DTD) paradigm unlike existing models. We introduce a new Monte Carlo (MC) scheme to synthesize realistic 6D DTD numerical phantoms and invert the MR signal. We show that the signal inversion is well-posed and estimate the CNTVD parameters parsimoniously by exploiting the different symmetries of the mean and covariance tensors of CNTVD. The robustness of the estimation pipeline is assessed by adding noise to calculated MR signals and compared with the ground truth. A family of invariant parameters and glyphs which characterize microscopic shape, size and orientation heterogeneity within a voxel are also presented.

## Introduction

Diffusion tensor imaging (DTI) is a widely accepted MR imaging method used by clinicians and neuroscientists to assess microstructure and organization of brain and other soft tissues^1,2^. DTI consists of measuring a single (mean) diffusion tensor within each voxel of an imaging volume and allows characterization of brain tissue microstructure, architecture, organization, and anatomical connectivity. Applications have been widespread in brain and whole body clinical imaging^3,4^. However, DTI only provides an estimate of a single mean diffusion tensor within each voxel, which is typically on the order of one cubic millimeter, representing a microliter of tissue. But, at microscopic, mesoscopic, and even macromolecular length scales the brain and other tissues contain a myriad of cell sizes and types, fine processes, extracellular matrix (ECM) and intracellular components with complex microstructural and architectural organizations^5^. Histological analysis often reveal distinct anisotropic domains within the same voxel, such as multiple white matter pathways which may interdigitate, kiss, cross, merge, diverge, etc. A goal of “in vivo MRI histology” or “microstructure imaging” has been to achieve subvoxel resolution and extract useful information about various salient water pools to provide a more nuanced and comprehensive assessment of the complex microscopic tissue milieu. Such information could be invaluable in understanding of normal and abnormal development, neuropathological changes, and brain structure–function relationships, to name a few.

The fundamental but coarse-scale description of diffusion anisotropy within a voxel, resulting from an anisotropic Gaussian diffusion process, is via DTI^1,2^. Several approaches were later introduced to capture both the heterogeneous and anisotropic character of diffusion within a MRI voxel such as the high angular resolution diffusion imaging (HARDI)^6^, generalized DTI using higher order tensors (HOT)^7^, and diffusion kurtosis imaging^8,9^. These models have well known limitations such as the unphysical increase in the diffusion weighted MR signal with b-value in the kurtosis model^10^ which limits the amount of microstructure information that could be gleaned from it given that DTI successfully accounts for most of the signal (approximately 90 percent) at low b-values^11^.

An important advance in the field was made by Jian et al. where the neural tissue is modeled to be composed of microscopic Gaussian diffusion compartments described by the joint probability distribution for the six elements of the diffusion tensor (i.e. diffusion tensor distribution or DTD) which in general is unknown^12^. Obtaining the 6D DTD non-parametrically involves taking the inverse Laplace transform (ILT) of the MR signal, which is a well-known ill-posed inverse problem^13^. To overcome the ill-posedness, Jian et al. assumed a discrete mixture of Wishart distribution for the DTD and showed it could resolve crossing fibers. Leow et al. reduced the DTD from 6D to 4D by assuming cylindrical symmetry and numerically computed it for the brain tissue^14^. The 4D DTD however has limited application in gray matter where the cylindrical symmetry assumption is not valid^15^. Walderhaug et al. introduced the cumulant expansion of MR signal for scalar diffusivity whereby the MR signal attenuation is approximated in terms of the moments of the DTD which are related to underlying microstructure^16^. The advantage of this method is that it does not assume a particular DTD but the expansion has a limited radius of convergence causing unphysical signal increase with b-value similar to the kurtosis model^10^. The cumulant description is also not compact in higher dimensions since the number of unknown parameters increases exponentially with increasing order of the HOT required to describe these moments thereby making it susceptible to over-fitting of the MR signal.

The aforementioned studies used rank-1 b-matrices resulting from their use of single pulsed-field gradient (sPFG) to obtain diffusion weighting, which limit the detection of microscopically anisotropic domains within a voxel^17,18^ which have been shown to exist in the brain and spinal cord^19–21^. Recently, Topgaard et al. obtained cylindrically symmetric 4D DTD in a phantom using higher rank b-matrix measurements with 2-step Monte Carlo (MC) signal inversion^22–24^. A key aspect of their work is to classify the underlying microstructure in terms of size, shape and orientation heterogeneity. It should be noted that estimating the 4D DTD using this method is time consuming both in computation and MR acquisition. Reymbaut et al. used matrix-variate Gamma distribution as DTD to overcome the unphysical signal increase with *b*-value of kurtosis and cumulant models since it is by definition restricted to positive definite matrices, however this distribution is limited in the types of heterogeneties it could capture^25,26^. Jespersen et al. used the cumulant expansion for orientationally averaged MR signal to detect microscopic diffusion anisotropy in brain tissue using rank-2 b-matrices^27,28^. Westin et al. extended the cumulant approach to 6D DTD with higher rank b-matrices and applied on the neural tissue for low diffusion weighting ($\text {b} < 2.5~\text {ms}/\mu \text {m}^2$) where the expansion is assumed to be valid^29^. The cumulant expansion was performed to include the first two moments of the DTD with heterogeneity measures derived from projections of the second moment (i.e., the covariance) with the isotropic tensor which often is not rich enough to describe the microstructural tissue complexity. The shape measure is sensitive to both shape and orientation heterogeneity while the orientation heterogeneity measure assume cylindrical symmetry of micro diffusion tensors which limit their applicability to complex neural tissue.

In this work, a novel paradigm is introduced to obtain the 6D-DTD within a voxel which overcomes the limitations discussed above. We assume the DTD to be a normal tensor-variate distribution^30^ but constrained to lie on the manifold of positive semi-definite diffusion tensors (CNTVD) which mitigate the unphysical signal increase with *b*-value in cumulant and kurtosis models while preserving the richness of DTD paradigm unlike the matrix-variate Gamma distribution and 4D DTDs. We also propose that the CNTVD is the natural and best choice for the DTD in the absence of *a priori* information about tissue microstructure due to its maximum entropy property. We simplify the experimental design by showing that rank-1 and rank-2 b-matrix measurements are sufficient for estimating the mean and covariance tensors of the DTD, and introduce a compressed sensing (CS) based sampling of b-matrices for time-efficient MR acquisition. We introduce a means to synthesize realistic diffusion tensors drawn from a given DTD, and a new MC scheme which is faster and simpler compared to the 2-step MC method in^22^ to invert the MR signal. We also introduce a parsimonious model selection framework exploiting the symmetries of the mean^31^ and covariance tensors^32^ to robustly estimate the CNTVD parameters rather than assuming a particular symmetry. Finally we introduce a family of new DTD derived stains and glyphs which characterize various aspects of tissue microstructure such as the micro-orientation distribution function ($\mu$ODF), micro-fractional anisotropy ($\mu$FA), and size, shape and orientation heterogeneity stains which are more general than that described by an isotropic covariance tensor and overcomes the limitations of previously defined metrics by independently capturing the desired heterogeneity.

## Methods

### MR signal model

The MR signal from an ensemble of diffusion tensors distributed according to $p({\mathbf {D}})$ is given by^29^,

1 $$\begin{aligned} S({\mathbf {b}}) = S_0 \int p({\mathbf {D}}) \exp \left( -{\mathbf {b}}^{\top } {\mathbf {D}}\right) d{\mathbf {D}} \end{aligned}$$

where $S_0$ is the signal without diffusion weighing, ${\mathbf {D}} = \left( D_{xx},D_{yy},D_{zz},D_{xy},D_{xz},D_{yz}\right) ^\top$ is a vector of the independent components of the second order symmetric diffusion tensor, and ${\mathbf {b}} = \left( b_{xx},b_{yy},b_{zz},2b_{xy},2b_{xz},2b_{yz}\right) ^\top$ is a vector of the independent components of the $3 \times 3$ symmetric diffusion weighting b-matrix^1^. For a normal distribution of tensors constrained in the manifold of positive definite tensors, ${\mathcal {M}}^+$, $p({\mathbf {D}})$ is given by,

2 $$\begin{aligned} p({\mathbf {D}}) = \frac{\exp \left[ -\frac{1}{2} ({\mathbf {D}}-\overline{{\mathbf {D}}})^{\top } \Omega ^{-1} ({\mathbf {D}}-\overline{{\mathbf {D}}})\right] {\mathbf {1}}_{{\mathcal {M}}^+}({\mathbf {D}})}{ \int _{{\mathbf {1}}_{{\mathcal {M}}^+}({\mathbf {D}})}\exp \left[ -\frac{1}{2} ({\mathbf {D}}-\overline{{\mathbf {D}}})^{\top } \Omega ^{-1} ({\mathbf {D}}-\overline{{\mathbf {D}}})\right] d{\mathbf {D}}} \end{aligned}$$

where ${\mathbf {1}}_{{\mathcal {M}}^+}({\mathbf {D}})$ is the indicator function and $\overline{{\mathbf {D}}}$ is second order mean diffusion tensor expressed as a $6 \times 1$ vector, and $\Omega$ is the fourth order covariance tensor, ${\mathsf {C}}$, expressed as a $6 \times 6$ matrix^32^ according to the following mappings,

3 $$\begin{aligned} \overline{{\mathbf {D}}}&= \left( \overline{{\mathsf {D}}}_{xx},\overline{{\mathsf {D}}}_{yy},\overline{\mathsf {D}}_{zz},\overline{\mathsf {D}}_{xy},\overline{\mathsf {D}}_{xz},\overline{\mathsf {D}}_{yz}\right) ^{\top } \end{aligned}$$

4 $$\begin{aligned} \Omega&= \left( \begin{array}{cccccc} {\mathsf {C}}_{xxxx} &{} {\mathsf {C}}_{xxyy} &{} {\mathsf {C}}_{xxzz} &{} {\mathsf {C}}_{xxxy} &{} {\mathsf {C}}_{xxxz} &{} {\mathsf {C}}_{xxyz} \\ {\mathsf {C}}_{xxyy} &{} {\mathsf {C}}_{yyyy} &{} {\mathsf {C}}_{yyzz} &{} {\mathsf {C}}_{yyxy} &{} {\mathsf {C}}_{yyxz} &{} {\mathsf {C}}_{yyyz} \\ {\mathsf {C}}_{xxzz} &{} {\mathsf {C}}_{yyzz} &{} {\mathsf {C}}_{zzzz} &{} {\mathsf {C}}_{zzxy} &{} {\mathsf {C}}_{zzxz} &{} {\mathsf {C}}_{zzyz} \\ {\mathsf {C}}_{xxxy} &{} {\mathsf {C}}_{yyxy} &{} {\mathsf {C}}_{zzxy} &{} {\mathsf {C}}_{xyxy} &{} {\mathsf {C}}_{xyxz} &{} {\mathsf {C}}_{xyyz} \\ {\mathsf {C}}_{xxxz} &{} {\mathsf {C}}_{yyxz} &{} {\mathsf {C}}_{xzzz} &{} {\mathsf {C}}_{xyxz} &{} {\mathsf {C}}_{xzxz} &{} {\mathsf {C}}_{xzyz} \\ {\mathsf {C}}_{xxyz} &{} {\mathsf {C}}_{yyyz} &{} {\mathsf {C}}_{zzyz} &{} {\mathsf {C}}_{xyyz} &{} {\mathsf {C}}_{xzyz} &{} {\mathsf {C}}_{yzyz} \end{array}\right) \end{aligned}$$

The signal equation is approximated using MC integration with samples, ${\mathbf {D}}_i$, drawn from the CNTVD as shown below,

5 $$\begin{aligned} S({\mathbf {b}}) \approx S_0 \left( \frac{ \sum _{i=1}^n \exp \left( -{\mathbf {b}}^{\top } {\mathbf {D}}_i\right) {\mathbf {1}}_{{\mathcal {M}}^+}({\mathbf {D}})}{ \sum _{i=1}^n {\mathbf {1}}_{{\mathcal {M}}^+}({\mathbf {D}})}\right) \end{aligned}$$

where *n* is the number of MC samples, set to 200,000 in our simulations. The choice of CNTVD for DTD is motivated by its resemblance to that measured in the brain tissue for trace of diffusion tensor^33^, and a few of its properties which we prove in the supplementary material. Given that DTD is generally unknown in a voxel, we show that CNTVD is the natural choice since it is the least informative distribution (or maximum entropy) among all probability densities supported by a constraint (e.g., positive definiteness of diffusion tensors) with a given first and second moments. This maximum entropy property of CNTVD avoids adding information that are not present^34^. We also prove that the moments of CNTVD are unique for a given DTD (Section 1 in the supplementary material) which can be estimated from the MR signal generated from rank-1 and rank-2 b-matrices (Section 4 in the supplementary material), which renders the data inversion problem well-posed.

For comparison, MR signal from the cumulant expansion and kurtosis model were also computed for the given mean and covariance. The kurtosis tensor, ${\mathsf {K}}_{ijkl}$, subsumed by the covariance tensor, is obtained by imposing additional symmetries on the covariance tensor resulting in the following identity (Section 2 in the supplementary material),

6 $$\begin{aligned} {\mathsf {K}}_{ijkl} = \frac{{\mathsf {C}}_{ijkl} + {\mathsf {C}}_{ikjl} + {\mathsf {C}}_{iljk}}{\left( \overline{{\mathsf {D}}_{xx} + {\mathsf {D}}_{yy} + {\mathsf {D}}_{zz}}\right) ^2} \end{aligned}$$

### DTD synthesis

The individual micro-diffusion tensors (i.e., MC samples) in the signal equation (Equation (5)) are synthesized from random samples drawn from 6D normal multivariate distribution with mean and covariance that define the desired DTD. The 6D samples are reformulated into $3 \times 3$ second order tensors using the equivalence between the two formulations shown by Basser and Pajevic^32^. A positive definiteness filter is applied on the drawn samples to satisfy the physical constraint on the diffusion tensor. In addition to obtaining the signal, the synthesized micro-diffusion tensors also demonstrate the richness of CNTVD.

Since the mean and covariance of a DTD is in general unknown *a priori*, it was calculated empirically from a sample of micro-diffusion tensors generated by randomly varying their eigenvalues and/or eigenvectors. For example, samples for an isotropic emulsion are generated from individual isotropic micro-diffusion tensors such that their trace follows a univariate normal distribution.

### Experimental design

It is impractical to span the entire space of positive semi-definite b-matrices of all three ranks. However, using tensor algebra we prove that a combination of rank-1 and rank-2 b-matrices are sufficient to span the entire space of fourth-order covariance tensors (Section 3 in the supplementary material). Further data reduction is achieved using CS which has been employed in several multi-dimensional MR studies exploiting the sparsity in *q*-space^35–37^.

**Figure 1:**
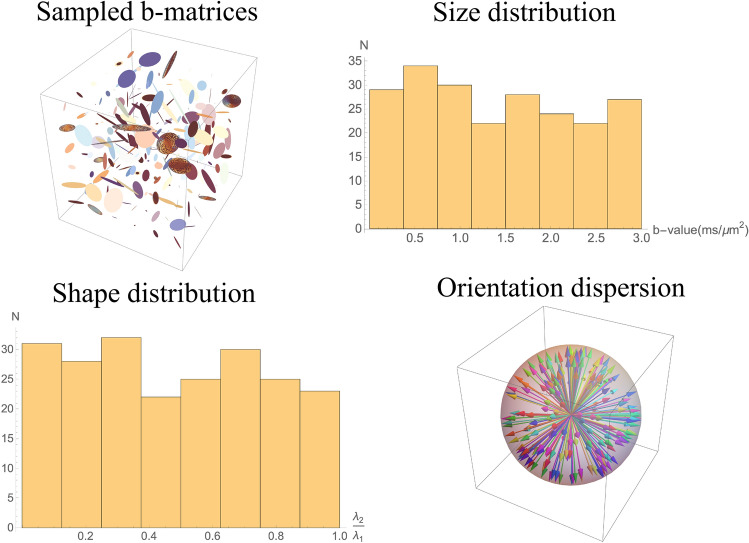
Experimental design for DTD estimation. (Top left) An example of the sampled rank-1 and rank-2 b-matrices (N = 216) shown as ellipses generated by randomly varying the sum and ratio of eigenvalues, and orientation of eigenvectors of the b-matrix. (Top right) Histogram of trace of b-matrix showing the uniform sampling in size. (Bottom left) Histogram of the ratio of two non-zero eigenvalues ($\lambda _1 > \lambda _2$) of the sampled b-matrices showing the uniform sampling in shape. (Bottom right) Orientation dispersion of the principal eigenvector of the sampled b-matrices showing the uniform sampling in orientation. The vectors are colored to distinguish the individual b-matrices. The above graphics was generated using the *Mathematica* software^67^.

A b-matrix of rank-*m*, ${\mathsf {b}}_m$, (with $m \in \{1, 2, 3\}$) is eigendecomposed as shown below,

7 $$\begin{aligned} {\mathsf {b}}_m&= {\mathsf {R}}\Lambda _m {\mathsf {R}}^\top \end{aligned}$$

8 $$\begin{aligned} {\mathsf {R}}&= {\mathsf {X}}(\alpha ) {\mathsf {Y}}(\beta ) {\mathsf {Z}}(\gamma ) \end{aligned}$$

9 $$\begin{aligned} \Lambda _m&= \begin{bmatrix} \lambda _1 &{} 0 &{} 0 \\ 0 &{} \ddots &{} 0 \\ 0 &{} 0 &{} \lambda _m \end{bmatrix} \end{aligned}$$

where ${\mathsf {X}}, {\mathsf {Y}}, {\mathsf {Z}}$ are the rotation matrices along *x*, *y*, *z* axes respectively, $\alpha ,\beta , \gamma$ are the Euler angles, and $\lambda _i$ is the $i^{th}$ the eigenvalue of the b-matrix. A set of rank-1 and rank-2 b-matrices are generated by randomly varying their eigenvalues such that their sum follow an uniform distribution within the range of b-values of interest for uniform sampling in size, and their ratios also follow an uniform distribution for uniform sampling in shape. Uniform sampling in orientation is achieved by randomizing the Euler angles and rotation order in the overall rotation matrix, ${\mathsf {R}}$. The resulting collection of b-matrices have varying orientation, size and shape, an example of which is shown in Fig. 1.

### Parameter estimation

For a given DTD, the MR signal is calculated for the designed b-matrices ($N = 216$) using Equation (5). Gaussian noise is added on the real and imaginary channels of the MR signal, if necessary, before inverting the resulting signal magnitude to obtain the CNTVD parameters characterizing the DTD. The number of unknowns for the most general DTD is 28 (6 for mean, 21 for the covariance and 1 for $S_0$), which makes the signal inversion susceptible to over-fitting^38^. A family of physically plausible nested models for the mean and covariance are then used to balance the bias and variance in the estimated parameters.

The mean tensor including $S_0$ is classified into the following four submodels: (1) 1-parameter noise model (i.e., only $S_0$), (2) 2-parameter isotropic model, (3) 5-parameter prolate/oblate models and (4) 7-parameter general anisotropic model^31^. The equivalence between the $6 \times 6$ covariance matrix and the fourth order tensor^32^ is used to classify the covariance tensor into the following eight submodels exploiting symmetries of the fourth order tensor well known in the elasticity literature^39^: (1) 2-parameter isotropic model, (2) 3-parameter cubic model, (3) 5-parameter hexagonal model, (4) 6/7-parameter trigonal model, (5) 6/7-parameter tetragonal model, (6) 9-parameter orthorhombic model, (7) 13-parameter monoclinic model, and (8) 21-parameter triclinic model.

The MR signal is successively fit to Equation (5) for the hierarchy of above nested models, ranging from scalar noise to DTI to the most complex 28-parameter DTD model (Section 5 in supplementary material), using a numerical optimization algorithm^40^ implemented in SciPy^41^ to estimate the unknown parameters. The Bayesian information criteria (BIC) is used for selecting models with greatest parsimony, (i.e., which provide an optimal trade-off between the goodness-of-fit and the number of free parameters)^38^. The model with the fewest parameters is selected unless the difference in its BIC with a more complex model is greater than two^42^. The appropriate mean tensor model is first selected from the data with the covariance set to zero to account for uniform DTDs. The selected mean tensor model is then augmented with each of the eight covariance models to find the one which parsimoniously describes the data.

### Microstructural stains and glyphs

The estimated CNTVD parameters are used to delineate several microstructural features within the voxel. The micro-diffusion tensors within the voxel are simulated by drawing samples from CNTVD with the estimated parameters. The mean tensor is visualized as an ellipsoid^2^, while the fourth order covariance tensor is visualized as a 3D glyph by projecting the tensor onto a 3D sphere using the tensor contraction, $r_i r_j r_k r_l {\mathsf {C}}_{ijkl}$, where ${\mathbf {r}}_{\theta ,\phi } = \left( \sin \theta \cos \phi , \sin \theta \sin \phi , \cos \theta \right)$ is the unit vector on a sphere^32,43^. The radius of the glyph along a given orientation is set to the projected value of the covariance in that direction. This glyph essentially shows the variability of the mean diffusion tensor as a function of orientation. Orientations along which the radial segments are large correspond to greater uncertainty whereas orientations along which radial segments are small correspond to lesser uncertainty.

Given the mean and covariance of CNTVD, microscopic quantities such as micro-fractional anisotropy ($\mu$FA), $\mu$ODF, micro-entropy (*S*) and $\mu$ODF entropy are computed using the following relation,

10 $$\begin{aligned} \mu f = \overline{f({\mathbf {D}})} \approx \frac{ \sum _{i=1}^n f\left( {\mathbf {D}}_i\right) \mathbf{1} ({\mathbf {D}}_i \in {\mathcal {M}}^+)}{ \sum _{i=1}^n \mathbf{1} ({\mathbf {D}}_i \in {\mathcal {M}}^+)} \end{aligned}$$

where *f* is the function of interest such as FA, ODF, overall entropy (i.e.$-\log p({\mathbf {D}})$) or ODF entropy, $S_{\text {ODF}}({\mathbf {D}})$, which is a measure of disorder in the orientation of diffusion tensors as shown below,

11 $$\begin{aligned} S_{\text {ODF}}({\mathbf {D}})&= - \int _0^{2\pi } \int _0^\pi \text {ODF}({\mathbf {D}},\theta , \phi ) \log \left( \text {ODF}({\mathbf {D}},\theta , \phi )\right) \sin \theta d\theta d\phi \end{aligned}$$

12 $$\begin{aligned} \text {ODF}({\mathbf {D}},\theta , \phi )&= \frac{1}{4\pi \sqrt{|{\mathbf {D}}_{3\times 3}|}\left( {\mathbf {r}}_{\theta ,\phi }^\top {\mathbf {D}}_{3\times 3}^{-1}{\mathbf {r}}_{\theta ,\phi }\right) ^{\frac{3}{2}}} \end{aligned}$$

where ${\mathbf {D}}_{3\times 3}$ is the diffusion tensor expressed in $3 \times 3$ matrix form, and $\text {ODF}({\mathbf {D}},\theta , \phi )$ is the ODF for diffusion tensor^44^. It should be noted that the above definition of $\mu$FA differ from previous definitions^28,29,45^ which either assume powder average of diffusion tensors or have difficulty interpreting them while our definition is more general and easy to interpret. In contrast to the scalar $\mu$FA quantity, the macro- and micro-ODFs within a voxel are displayed as 3D glyphs where the radius of the glyph along a given orientation is equal to the value of the ODF along that direction. The $\mu$ODF glyph shows the distinct coherent populations of diffusion tensors present within a voxel at the microscopic level.

The size heterogeneity within a voxel is expressed as the variance of mean ADC (mADC) or average trace of the individual micro diffusion tensors. Given a multivariate normal random variable, ${\mathbf {D}}$, it is well known that the distribution of a scalar projection of ${\mathbf {D}}$, ${\mathbf {C}}^{\top }{\mathbf {D}}$, where ${\mathbf {C}}$ is a $1 \times 6$ random constant vector, has mean, ${\mathbf {C}}^{\top }{\mathbf {D}}$, and variance, ${\mathbf {C}}^{\top } \Omega {\mathbf {C}}$^46^. Using this identity, variance of mADC is obtained by choosing ${\mathbf {C}}$ equal to $\frac{1}{3}(1,1,1,0,0,0)$. This results in the mADC variance equal to the arithmetic average of upper $3 \times 3$ block matrix appearing in $\Omega$ from which the expression for variation in size, $\text {V}_{\text {size}}$, is given below^47^,

13 $$\begin{aligned} \text {V}_{\text {size}} = \sqrt{\frac{1}{9} \sum _{i=1}^3\sum _{j=1}^3 \Omega _{ij}} \end{aligned}$$

It takes a value of zero for uniformly sized tensors and increases with increasing variability in the trace of the diffusion tensors within a DTD motif. Given the shape of an ellipsoid is uniquely determined by the ratio of its eigenvalues^48^, the shape heterogeneity within a voxel is expressed as the root sum of the variance of eigenvalue ratios of micro-diffusion tensors, $\text {V}_{\text {shape}}$, as given below,

14 $$\begin{aligned} \text {V}_{\text {shape}} = \sqrt{\text {Var}\left( \frac{\lambda _2}{\lambda _1}\right) + \text {Var}\left( \frac{\lambda _3}{\lambda _2}\right) } \end{aligned}$$

where $\lambda _1> \lambda _2 > \lambda _3$ are the individual eigenvalues of the micro-diffusion tensor and $\text {Var}$ is the variance. It takes a value of zero for uniformly shaped tensors and increases with increasing variability in the shape of the diffusion tensors within a DTD motif. The orientation heterogeneity within a voxel is expressed as the extent of orientation dispersion of eigenvectors within a DTD motif, $\text {V}_{\text {orient}}$, proposed in^49^,

15 $$\begin{aligned} \text {V}_{\text {orient}}&= \min _{i=1,2,3}\sqrt{\frac{\beta ^i_2 + \beta ^i_3}{2\beta ^i_1}} \end{aligned}$$

where $\beta ^i_1> \beta ^i_2 > \beta ^i_3$ are the eigenvalues of the mean second order dyadic tensor formed by averaging the outer products of the $i^{th}$ eigenvector of the micro-diffusion tensor with itself. It takes a value of zero for coherently oriented diffusion tensors and one for randomly oriented diffusion tensors irrespective of their size and shape. The efficacy of the proposed stains in capturing the desired heterogeneity were evaluated by discretely and continuously mixing populations of diffusion tensors of varying size, shape and orientation.

## Results

**Figure 2:**
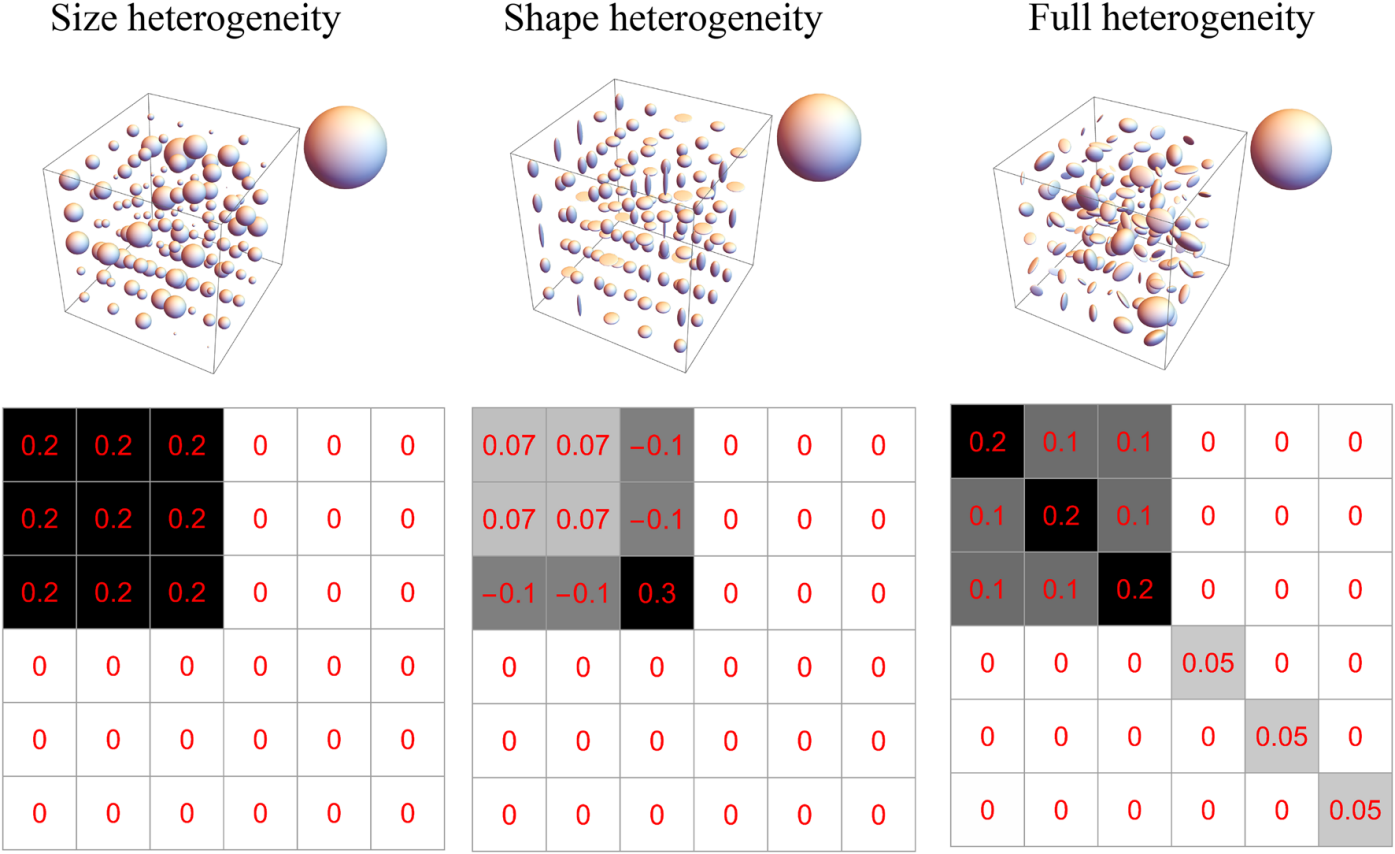
Macroscopically isotropic and microscopically heterogeneous DTDs generated from CNTVD describing gray matter. The DTD along with the mean diffusion tensor ellipsoid (inset) is shown in the top row while the structure of the $6 \times 6$ covariance matrix with non-zero elements highlighted is shown in the bottom row. (Left column) Emulsion type DTD with size heterogeneity, (Middle column) Shape heterogeneous DTD with a mixture of prolate, oblate and spherical diffusion tensors, (Right column) Fully heterogeneous DTD with randomly oriented tensors of varying shapes and sizes. The above graphics was generated using the *Mathematica* software^67^.

**Figure 3:**
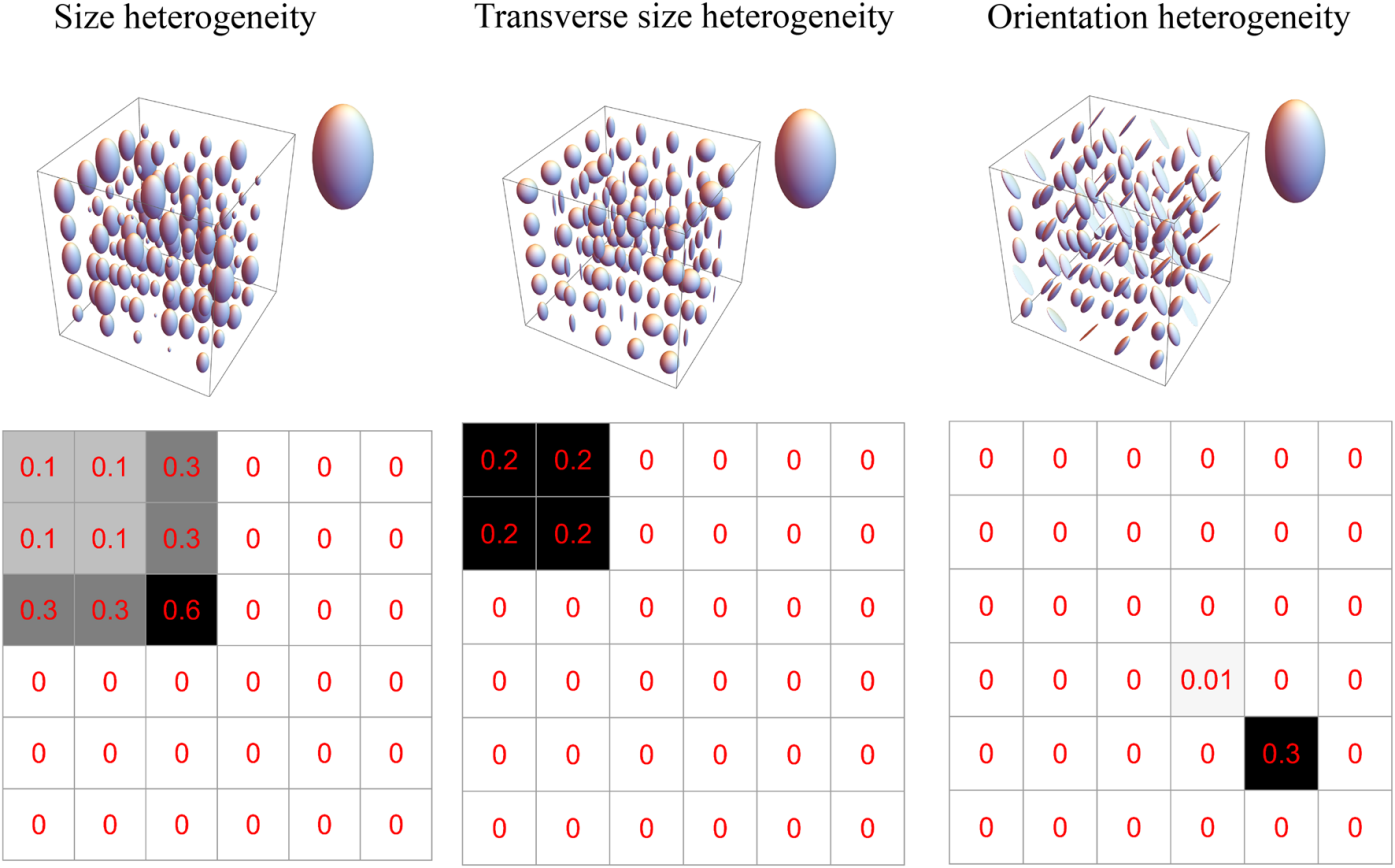
Macroscopically anisotropic and microscopically heterogeneous DTDs generated from CNTVD describing the white matter. The DTD along with the mean diffusion tensor ellipsoid (inset) is shown in the top row while the structure of the $6 \times 6$ covariance matrix with non-zero elements highlighted is shown in the bottom row. (Left column) Emulsion type DTD with size heterogeneity, (Middle column) DTD with random transverse eigenvalues characterizing a bundle of white matter fibers of varying diameters, (Right column) Crossing fiber DTD with 90-degree angle between the fibers. The above graphics was generated using the *Mathematica* software^67^.

A sample of microstructural templates representative of white and gray matter voxels generated using CNTVD is shown in Figs. 2 and 3. These figures shows the ability of CNTVD to remove the ambiguity of DTI model by assigning distinct covariance matrices corresponding to each of these DTDs even though their mean diffusion tensors are identical (also shown in the figures). The obtained covariance matrices displayed a wide range of symmetries not restricted to it’s most general form with 21 independent components. The figures also show how a single covariance matrix could describe a mixture of size, shape and orientation heterogeneities that maybe present within a MRI voxel.

A gray matter voxel is modeled as macroscopically isotropic but microscopically composed of diffusion tensors of varying size, shape and orientation as shown in Fig. 2. The size and fully heterogeneous (i.e., size, shape and orientation heterogeneous) DTDs exhibited an isotropic covariance structure characterized by two constants, $\lambda$ and $\mu$, similar to the bulk and shear modulus of the isotropic elasticity tensor^30,32^. The size heterogeneous DTD had $\lambda \ne 0$ and $\mu = 0$ while fully heterogeneous DTD had $\lambda \ne 0$ and $\mu \ne 0$. The shape heterogeneous DTD exhibited a hexagonal covariance structure.

A white matter voxel is modeled as macroscopically anisotropic and microscopically composed of distinct anisotropic subdomains shown in Fig. 3. The three DTDs considered are anisotropic emulsion (size heterogeneity), an axon bundle consisting of varying diameter fibers modeled by randomly varying the transverse eigenvalues together but fixing the longitudinal eigenvalue of the micro-diffusion tensors (transverse size heterogeneity), and two fibers crossing at 90 degrees (orientation heterogeneity). The crossing fiber exhibited an orthorhombic covariance structure while the other two DTDs exhibited a hexagonal structure.

**Figure 4:**
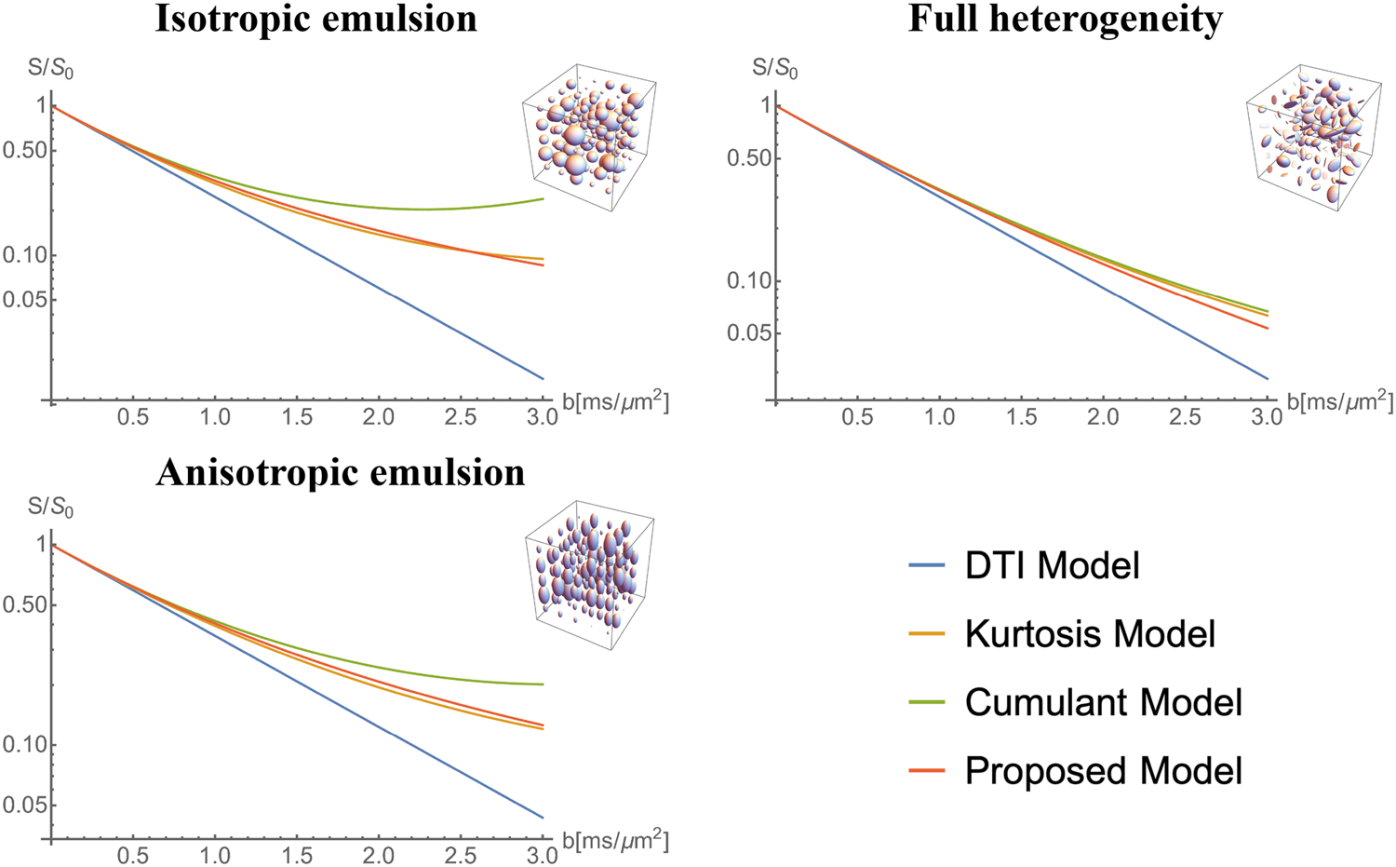
Comparison of signal models (DTI, kurtosis, second order cumulant and proposed model) for DTDs shown in the inset generated from CNTVD. Normalized MR signal $S/S_0$ is plotted for various b-values from rank-2 b-matrices with fixed shape and orientation. The above graphics was generated using the *Mathematica* software^67^.

The MR signal for some of the aforementioned DTDs obtained using the proposed model is compared with DTI, kurtosis models and second order cumulant approximation in Fig. 4. Since $p({\mathbf {D}})$ for these DTDs is the CNTVD, the proposed model is the ground truth for the MR signal. The kurtosis model and cumulant approximation in addition to the known unphysical signal increase with b-value begins to deviate from the ground truth at very low b-values well before the minimum in the signal decay curve occurs; thus cumulant approximation and kurtosis model are only valid for very low b-values where the DTI model is dominant.

**Figure 5:**
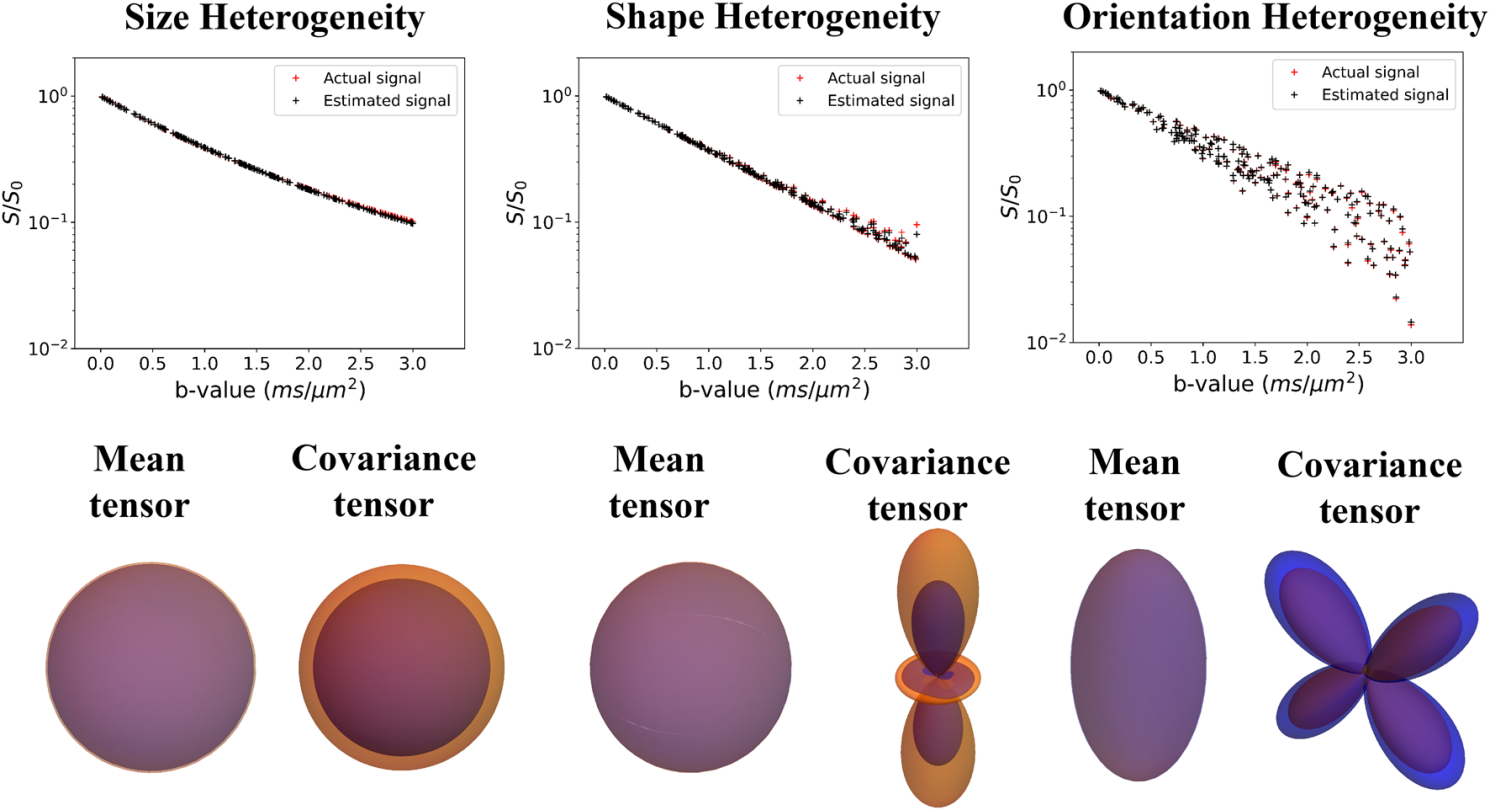
Estimation of mean and covariance tensors from synthetic MR signal without noise. The estimation is shown for three different DTDs (isotropic emulsion, shape heterogeneous and crossing fiber type DTDs) shown in Figs. 2 and 3. The signal curve using the proposed model obtained from actual and estimated mean/covariance tensors is shown in the first row. The mean and covariance glyphs for actual (orange) and estimated (blue) DTD are overlaid and shown in the second row. The above graphics was generated using the *matplotlib* package^68^, and the *Mathematica* software^67^.

The well-posedness of the signal inversion problem is demonstrated in Fig. 5 for various DTDs with clinically feasible b-values. The figure compares the actual signal with that calculated using the estimated parameters in the absence of noise. The error between the actual and estimated mean and covariance tensors are quantified using Frobenius norm in Table 1. For our demonstration we chose isotropic emulsion, shape heterogeneous, and crossing fiber type DTDs. The estimated and actual mean/covariance glyphs are overlaid for comparison. The estimation framework was able to capture all the tested mean–covariance pairs with errors less than 30% likely due to convergence criterion of the optimization algorithm and/or choice of initial parameter estimate.

**Table 1:** Performance of the estimation pipeline and heterogeneity stains evaluated using different signal-to-noise ratio (SNR) for three different DTDs.

DTD type	SNR	Estimation error in mean tensor (%)	Estimation error in covariance tensor (%)	Error in $\text {V}_{\text {size}}$ (%)	Error in $\text {V}_{\text {shape}}$ (%)	Error in $\text {V}_{\text {orient}}$ (%)
Size heterogeneity	5	4.0	30.0	14.9	–	–
	10	4.0	30.0	14.9	–	–
	20	1.0	5.0	2.1	–	–
	$\infty$	2.0	10.0	6.4	–	–
Shape heterogeneity	5	2.0	60.0	–	40.0	–
	10	1.0	50.0	–	40.0	–
	20	1.0	30.0	–	17.5	–
	$\infty$	0.6	30.0	–	15.0	–
Orientation heterogeneity	5	1.0	30.0	–	–	24.1
	10	0.8	20.0	–	–	20.7
	20	0.8	20.0	–	–	20.7
	$\infty$	0.8	20.0	–	–	20.7

Size heterogeneity refers to isotropic emulsion type DTD, shape heterogeneity refers to shape heterogeneous DTD with isotropic mean diffusion tensor and orientation heterogeneity refers to the DTD with two fibers crossing at 90$^\circ$. The estimation error in mean and covariance tensors are calculated using the Frobenius norm of the difference between the actual and estimated tensors divided that of the actual tensor and expressed as percentage. The error in stains (i.e. $\text {V}_{\text {size}},\text {V}_{\text {shape}}, \text {and}~\text {V}_{\text {orient}}$) were expressed as percent change from the ground truth value.

**Figure 6:**
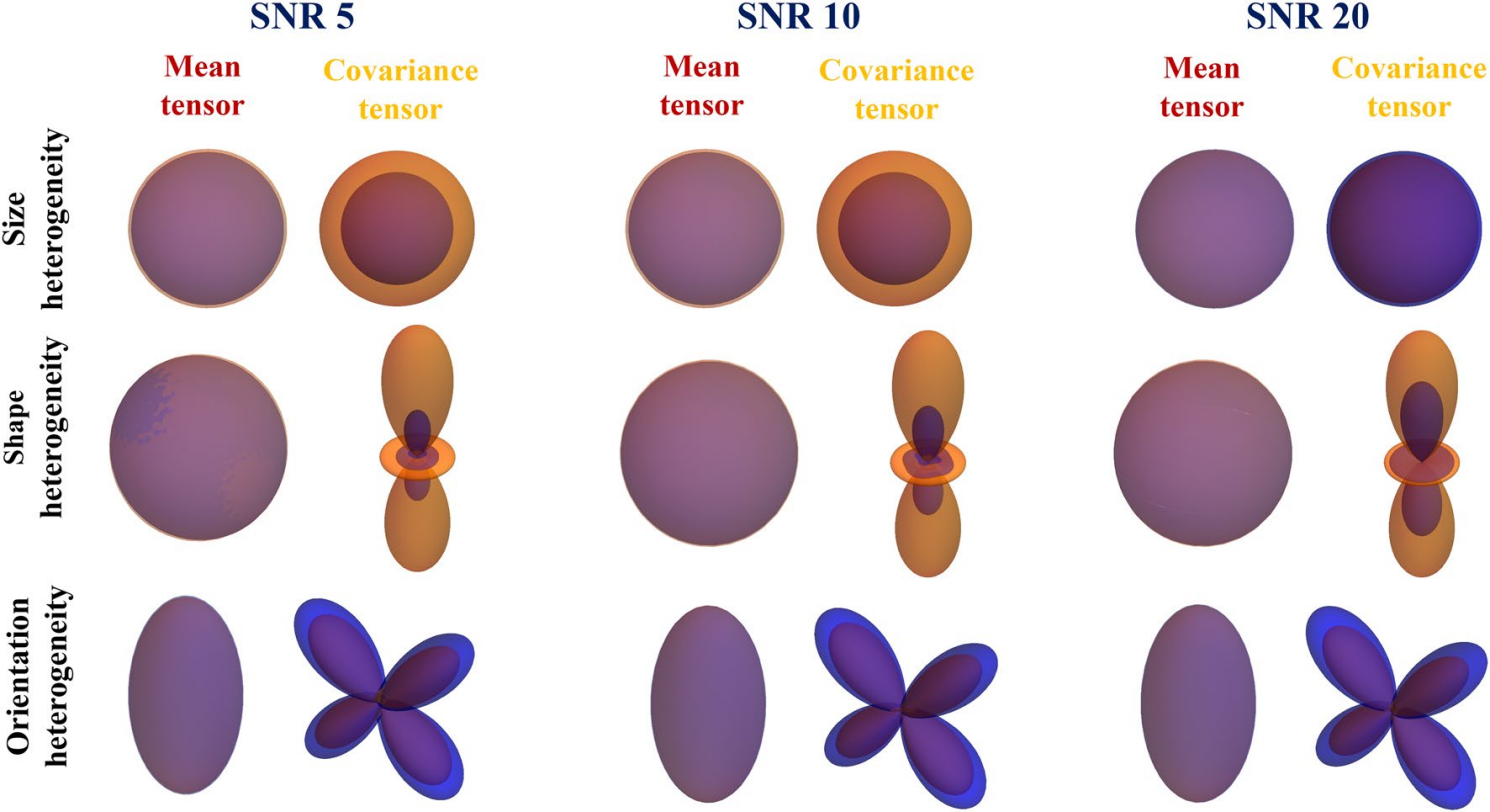
Estimation of mean and covariance tensors from synthetic MR signal for different DTDs with varying signal-to-noise ratio (SNR) at the largest b-value. The mean and covariance glyphs for actual (orange) and estimated (blue) DTD are overlaid. (First row) Isotropic emulsion, (Second row) Shape heterogeneous DTD and (Third row) Crossing fiber type DTD. The reduction in covariance estimation error with increasing SNR is clearly visible in shape and orientation heterogeneous DTD. The above graphics was generated using the *Mathematica* software^67^.

The robustness of the estimation pipeline to noise in the MR signal is shown graphically in Fig. 6, and quantified in Table 1. Three different realistic signal-to-noise ratios (SNR at largest b-value) are tested for the same DTDs as in the previous figure, with the estimated and actual mean/covariance glyphs overlaid for comparison. The mean tensor shape and size was exactly captured for all the SNRs. The structure of the covariance was also estimated accurately for all the three SNRs. However, there were errors in the size of the glyph and heterogeneity stains which reduced with increasing SNR for all the DTDs as shown in Table 1.

**Figure 7:**
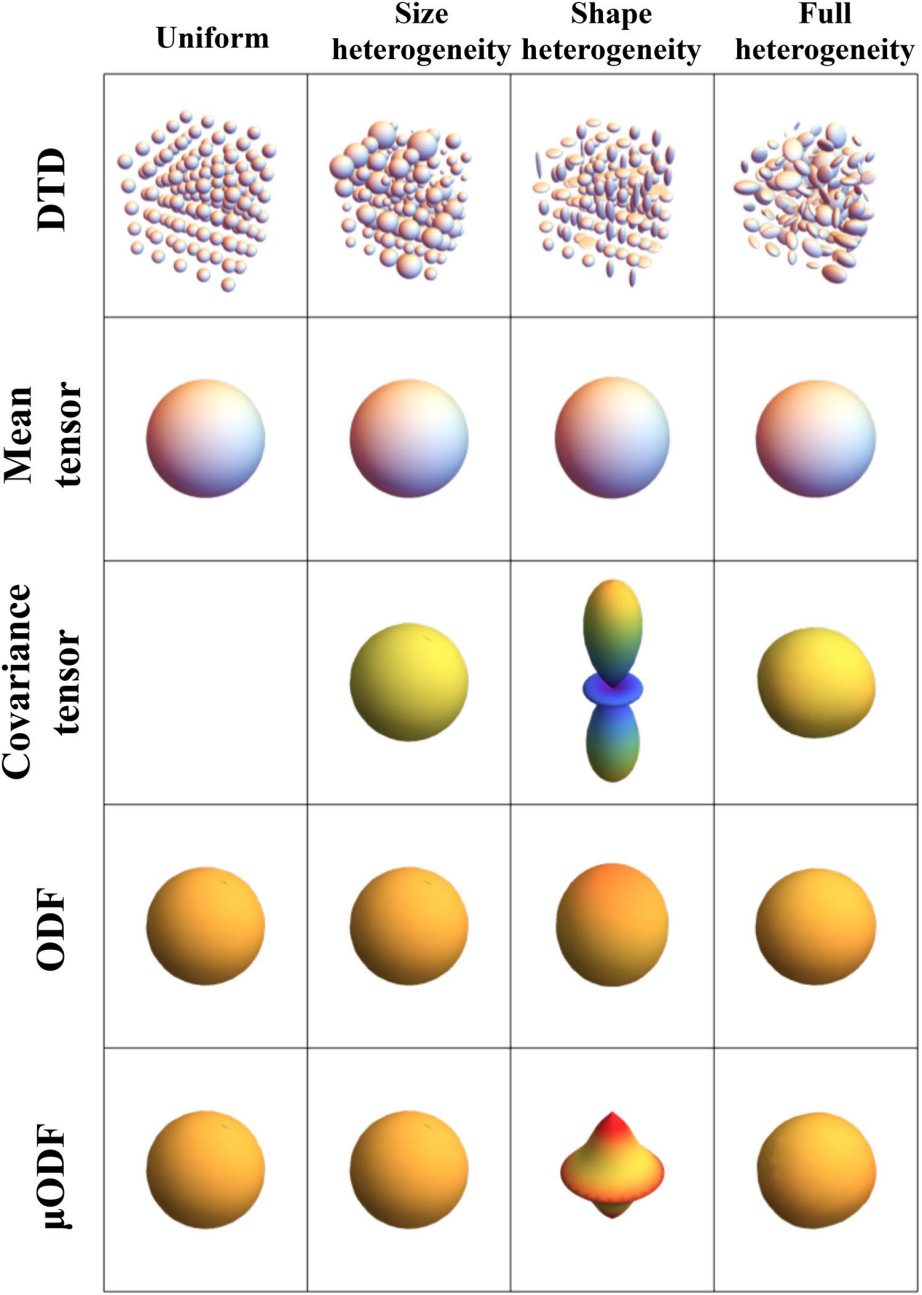
Glyphs describing macroscopically isotropic and microscopically heterogeneous DTDs generated from CNTVD. (First row) DTDs describing uniform, size, shape and orientation/size/shape heterogeneous voxels respectively. (Second row) Second order mean tensor displayed as an isosurface for each of the DTDs. (Third row) Fourth order covariance tensor projected in 3D for each of the DTDs. (Fourth row) Macro ODF obtained from the mean diffusion tensor. (Fifth row) Micro ODF obtained by averaging the ODFs of individual micro diffusion tensors in the voxel. The above graphics was generated using the *Mathematica* software^67^.

**Figure 8:**
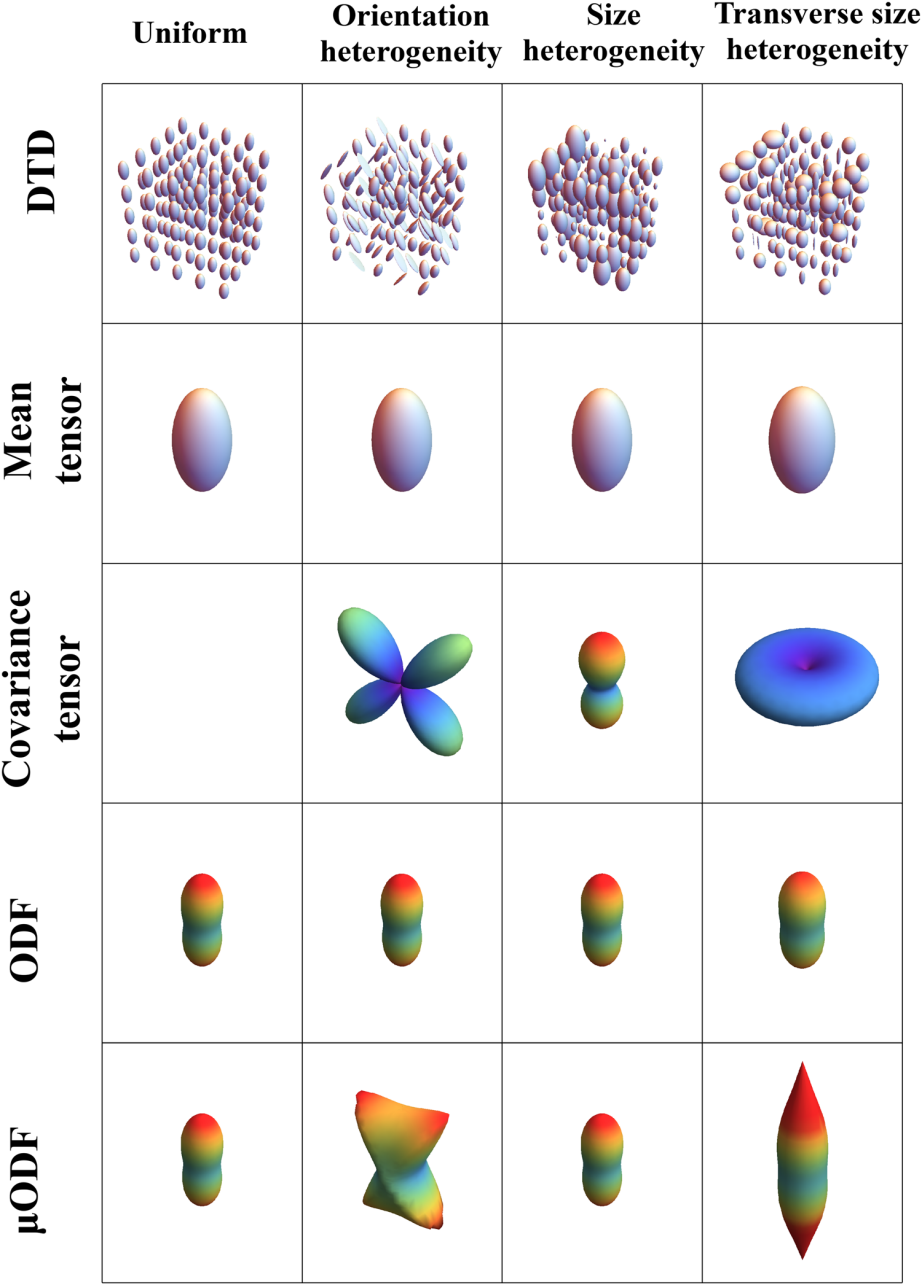
Glyphs describing macroscopically anisotropic and microscopically heterogeneous DTDs generated from CNTVD. (First row) DTDs describing uniform, orientation, size, and transverse size heterogeneous voxels respectively. (Second row) Second order mean tensor displayed as an iso-surface for each of the DTDs. (Third row) Fourth order covariance tensor projected in 3D for each of the DTDs. (Fourth row) Macro ODF obtained from the mean diffusion tensor. (Fifth row) Micro ODF obtained by averaging the ODFs of individual micro diffusion tensors in the voxel. The above graphics was generated using the *Mathematica* software^67^.

**Figure 9:**
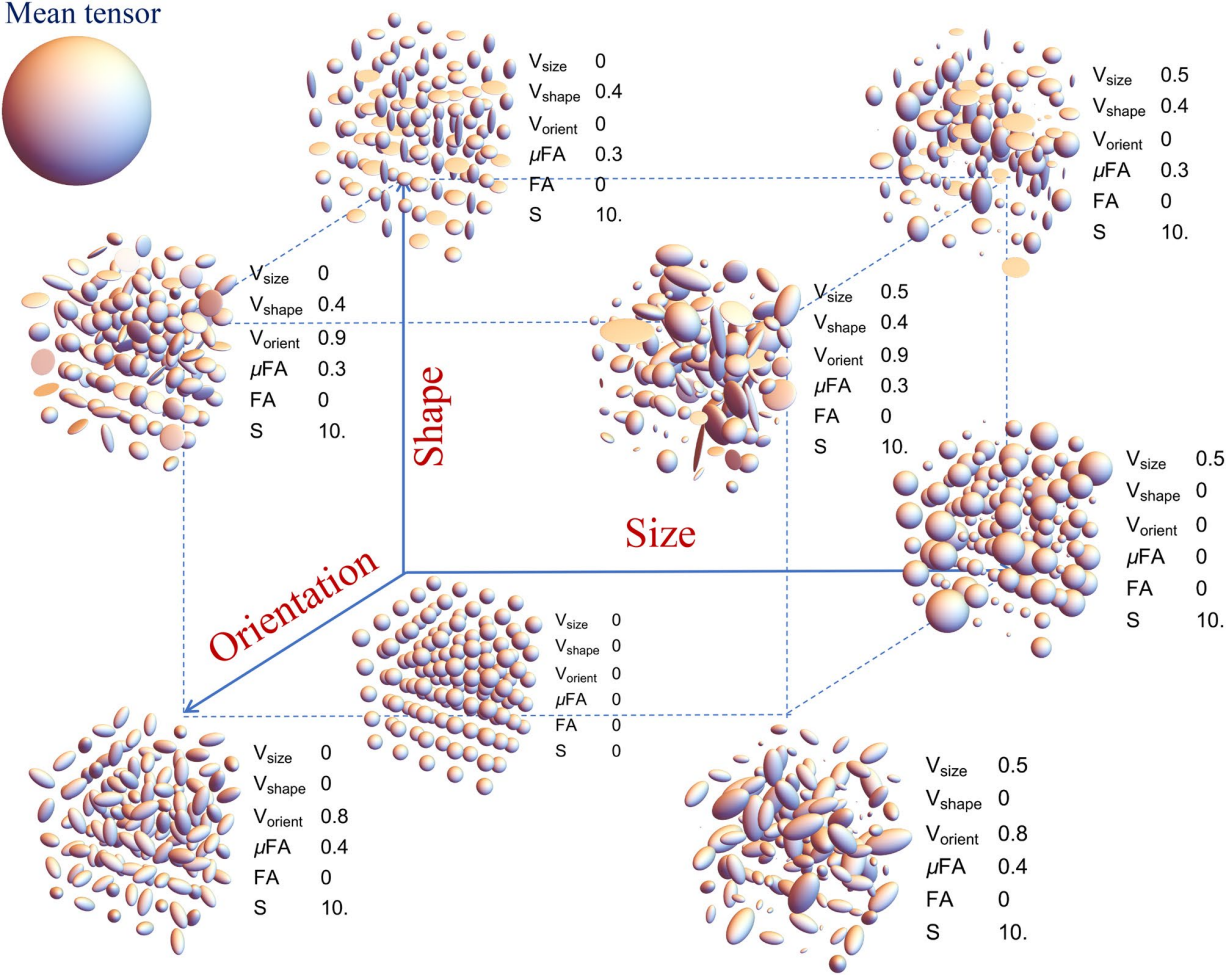
Various parametric stains depicting the shape, size and orientation heterogeneity for a macroscopically isotropic mean diffusion tensor shown on the upper left corner. The DTD and calculated values of the various stains are shown at various points in the 3D grid. Size axis refers to heterogeneity in the trace of the diffusion tensors, shape axis refers to heterogeneity in the shapes of the diffusion tensors, and the orientation axis refers to the heterogeneity in the orientation of the diffusion tensors. $\text {V}_{\text {size}}$ —stain quantifying the variation in the size (i.e. trace) of the diffusion tensors, $\text {V}_{\text {shape}}$ -—stain quantifying the variation in the shape of diffusion tensors, $\text {V}_{\text {orient}}$ —stain quantifying the orientation dispersion of diffusion tensors, $\mu \text {FA}$ —microscopic fractional anisotropy, $\text {FA}$ —fractional anisotropy, and $\text {S}$ —overall entropy of the DTD. The above graphics was generated using the *Mathematica* software^67^.

The various DTDs are visualized using glyphs and microstructural stains in Figs. 7,8 and 9. The glyphs for gray matter DTDs are shown in Fig. 7. The uniform DTD has an isotropic mean tensor, zero covariance and isotropic macro and micro ODFs as expected. Size and fully heterogeneous DTDs had the same glyphs as the uniform DTD except for the covariance, which was nonzero and isotropic. The covariance tensor glyph of shape heterogeneous DTD peaked along the longitudinal axis because of the large change in the eigenvalues that occur along that direction with eigenvectors fixed. It’s macroODF was isotropic while the $\mu$ODF was top shaped distinguishing the two dominant populations of tensors in the DTD (i.e., prolate and oblate tensors).

The glyphs for white matter DTDs are shown in Fig. 8. The mean tensor was prolate shaped, and macroODF was peanut shaped for all the DTDs considered. The structure of the covariance tensor was however unique for each DTD. The covariance tensor and $\mu$ODF for the crossing fibers had four lobes aligned with the two fiber populations. The covariance and $\mu$ODF for emulsion type DTD had two lobes along the principal direction of the mean diffusion tensor. The covariance for the multi diameter fiber bundle was pancake shaped showing the heterogeneity in the transverse plane. The $\mu$ODF was pill shaped due to hindered diffusion heterogeneity in the transverse plane compared to the longitudinal axis.

The calculated values of the proposed microstructural stains for a variety of DTDs with an isotropic mean diffusion tensor are shown graphically in Fig. 9. The $\mu$FA was greater than FA only for shape and/or orientation heterogeneous DTD hence it fails to capture the size heterogeneity and less specific. The entropy measure was overall higher for size, shape and orientation heterogeneous DTDs compared to the uniform DTD. The size heterogeneity stain, $\text {V}_{\text {size}}$, is zero for shape and orientation heterogeneous DTD but non-zero for size heterogeneous DTD. The shape heterogeneity stain, $\text {V}_{\text {shape}}$, is zero for size and orientation heterogeneous DTDs but non-zero for shape heterogeneous DTD. The orientation stain, $\text {V}_{\text {orient}}$, is zero for size and shape heterogeneous DTDs but non-zero for orientation heterogeneous DTD. Results from continuous mixing of populations of diffusion tensors can be found in Section 6 in the supplementary material which further show the ability of the proposed stains to independently capture the size, shape and orientation heterogeneities present within a voxel.

## Discussion

### CNTVD for DTD

The various white and gray matter DTDs demonstrate the richness of CNTVD in describing a variety of realistic sub-domains that may exist in a voxel in the brain. For a given mean diffusion tensor, these simulated DTDs also show that CNTVD could capture multiple types of heterogeneity that maybe present within a MRI voxel. However, it should be noted that the DTD in a voxel is generally unknown, and CNTVD is proposed as one of the admissible candidate distributions for its biological resemblance in brain tissue along with useful properties such as well-posed signal inversion, maximum entropy and producing positive definite diffusion tensors. Despite the richness of the CNTVD, it however does not span the entire space of positive semi-definite diffusion tensors. CNTVD for example could not describe situations with only orientational heterogeneity (i.e., a powder pattern) which is known to have DTD moments greater than two^50^. Simulations showed that CNTVD could also not capture crossing fibers with angular separations not equal to 90 degrees which require a mixture type model with multiple mean and covariance matrices. It should be noted that the presented framework could be applied to any DTD not restricted to CNTVD.

### Signal model

The signal curves show the need for large b-value data to observe the effects of intravoxel heterogeneity given DTI model successfully accounts for the signal at small b-values. The large b-values also sensitizes the signal to multiple DTD compartments such as intra- and extra-cellular environments which can be captured by expressing the proposed signal equation as a weighted sum of multiple DTDs. With the above modification, the limiting b-value for our proposed model is determined by the extent of diffusion independent signal loss such as due to MR relaxation and proton density unlike the cumulant expansion and kurtosis model.

The versatility of the proposed signal model is demonstrated by its validity at large b-values excluded by the kurtosis model and cumulant approximation, and for any $p({\mathbf {D}})$ not restricted to CNTVD. However, a central assumption of the model is the Gaussian diffusion of water within the tissue micro-compartments. A classic test for Gaussian diffusion is the time independence of diffusivity. The time-dependent diffusion in neural tissue is however experimentally observed only at very short diffusion times on the order of hundreds of microseconds^51^. The change in diffusivity in gray and white matter with diffusion times beyond a few milliseconds is very small ($< 10\%$ change from tens of milliseconds to two seconds)^52–54^. Further the breakdown of rotational invariance property of trace of the diffusion tensor, a known consequence of time-dependent diffusion^55^, was also found to be very small ($<5\%$) in neural tissue^56^. The apparent time independent diffusion at long diffusion times typically probed in diffusion MRI experiments^57,58^ could be partly attributed to pore saturation of wide diffusion gradient pulses, water permeability of lipid membranes which hinders the diffusion rather than restricting it, and/or approach to Gaussianity due to ensemble averaging of microscopic diffusion environments within a MRI voxel. The currently available data thus seems to suggest that at time scales of 20–60 ms typically probed in standard spin echo (SE) diffusion weighted scans, the water diffusion in the brain parenchyma is approximately Gaussian.

### Experimental design

The estimation pipeline was accurate and robust to noise which is partly attributed to the model selection framework and the novel experimental design which uniformly samples b-matrices of varying size, shape and orientation. The equivalence between the covariance matrix and fourth order covariance tensor representation introduced in^32^ enabled us to exploit the symmetries of the general fourth order tensor to build a family of nested models for covariance estimation.

The importance of the choice of diffusion encoding gradient vectors in the estimation of the diffusion tensor has been well studied^59,60^. It can be expected that the same holds true for DTD estimation as well especially for higher rank b-matrix measurements requiring multiple gradient pulses which increases the number of degrees of freedom. An optimal experimental design which significantly reduces the number of measurements is essential for practical applicability of the method. Komlosh et al. used rank-2 b-matrices obtained using orthogonal gradient pairs to extract microscopic heterogeneity in excised spinal cord^20^. Jespersen et al. introduced a *t*-design scheme for rank-2 b-matrix measurements to estimate powder averaged DTDs in the excised brain^28^. The two gradient vectors were made orthogonal to each other and chosen as the vertices of the icosahedron. The orthogonality constraint on the gradient vectors restricts the space of positive definite matrices explored, while sampling antipodal vectors leads to redundant b-matrices which ill-conditions the DTD signal inversion. Martins and Topgaard uniformly sampled the space of orientation, shape and size of the b-matrices in a phantom experiment which is extremely time consuming^24^. Westin et al. and Nilsson et al. used a multi-shell approach where each shell is composed of a number of rank 1, 2 and 3 b-matrices to account for arbitrary DTDs in the brain and reduce acquisition time in-vivo^29,61^.

Our experimental design using CS balances acquisition time and the space of fourth order covariance tensors spanned by the b-matrices^62^. A continuum of b-matrices of varying shapes, sizes and orientations were chosen instead of a multi-shell acquisition to get a better definition of the MR signal dependence with b-value. Such an experimental design allows an unbiased estimation of the DTD parameters in a reasonable amount of time. The lack of an extra gradient pair for rank-3 b-matrices, which were shown to be not necessary to estimate the covariance, reduces the number of degrees of freedom to explore and possibly the echo time of the acquisition particularly in clinical MRI scanners which have long gradient ramp times. It should be noted that the number of b-matrices and the range of b-values required to estimate the DTD parameters largely depend on the sample. The sampled b-value should be large enough such that residual in the data unaccounted by the DTI model lies well above the noise floor but small enough to prevent sensitization of multiple tissue compartments. The number of b-matrices is inversely proportional to the amount of residual in the data unaccounted by the DTI model for adequate statistical power.

### DTD visualization

The presented glyphs and stains provide an unique information about the underlying micro structure as shown in the figures. The $\mu$ODF glyph disentangled the coherent diffusion tensor populations present within a voxel as shown in shape heterogeneous and crossing fiber DTDs. The covariance glyph displayed the plane of maximal heterogeneity as shown in transverse size heterogeneous DTD. We show that the $\mu$FA does not fully capture the heterogeneity in the tissue thus the need for additional stains. It cannot distinguish between orientation and shape heterogeneities. Using simulations, we have also shown that our new stains independently capture the size, shape and orientation heterogeneities within the voxel overcoming some of the limitations in previous works^29^.

The proposed stains are generic and applies to any of the eight covariance models. They could help inform about normal and abnormal micro structural changes in tissue with disease. For Gaussian diffusion, the ODF glyph purely characterizes the orientation dependence of diffusivity independent of experimental parameters such as diffusion time. Comparing the macro and micro ODF glyphs could be particularly revealing in white matter voxels. The size metric could help distinguish between layers of the cerebral cortex which are known to have different cell size distribution that gets altered in diseases such as schizophrenia and Huntington’s disease^63^. It could also reveal the cytoarchitecture in spinal cord where diameter distribution of various fiber tracts and nerve roots traversing the cord vary widely^64–66^.

## Conclusion

In this study, a novel paradigm to simulate and estimate DTD is presented. A new MC based signal model is introduced which is monotonically decreasing with b-value unlike previous models and designed to work for any DTD. The richness of CNTVD is shown using a set of realistic DTDs that may exist in brain gray and white matter. The data inversion is performed using a parsimonious approach to accurately estimate various mean and covariance pairs in the presence of noise in the MR signal. A family of new novel parametric stains and glyphs are shown to capture distinct features of micro structural inhomogeneity within a MRI voxel. The method is thus an important advance in the continuing quest to explore the microstructure within the MRI voxel.

## Supplementary information

Supplementary material 1

## References

[1] Basser PJ, Mattiello J, Denis LB (1994). Estimation of the effective self-diffusion tensor from the NMR spin echo. J. Magn. Reson. Ser. B.

[2] Basser PJ, Mattiello J, LeBihan D (1994). MR diffusion tensor spectroscopy and imaging. Biophys. J ..

[3] Basser Peter J, Pajevic S, Pierpaoli C, Duda J, Aldroubi A (2000). In vivo fiber tractography using DT-MRI data. Magn. Reson. Med..

[4] Kenkel D, Spiczak J, Wurnig Moritz C (2016). Whole-body diffusion tensor imaging. J. Comput. Assist. Tomogr..

[5] Kasthuri N, Hayworth Kenneth J, Berger Daniel R (2015). Saturated reconstruction of a volume of neocortex. Cell.

[6] Tuch, D. S., Weisskoff, R. M., Belliveau, J. W. & Wedeen, V. J. High Angular Resolution Diffusion Imaging of the Human Brain. In: 321 (Philadelphia, 1999).

[7] Özarslan E, ThomasH M (2003). Generalized diffusion tensor imaging and analytical relationships between diffusion tensor imaging and high angular resolution diffusion imaging. Magn. Reson. Med..

[8] Liu C, Bammer R, MichaelE M (2003). Generalized diffusion tensor imaging (GDTI): A method for characterizing and imaging diffusion anisotropy caused by non-Gaussian diffusion. Isr. J. Chem..

[9] Jensen Jens H, Helpern Joseph A, Ramani A, Lu H, Kaczynski K (2005). Diffusional kurtosis imaging: The quantification of non-Gaussian water diffusion by means of magnetic resonance imaging. Magn. Reson. Med..

[10] Kiselev VG, Il’yasov KA (2007). Is the “biexponential diffusion” biexponential?. Magn. Reson. Med..

[11] Basser PJ (2002). Relationships between diffusion tensor and q-space MRI. Magn. Reson. Med..

[12] Bing J, Vemuri Baba C, Ozarslan E, Carney Paul R, Mareci Thomas H (2007). A novel tensor distribution model for the diffusion-weighted MR signal. NeuroImage.

[13] Callaghan PT (2011). Translational Dynamics and Magnetic Resonance.

[14] Leow AD, Zhu S, Zhan L (2009). The tensor distribution function. Magn. Reson. Med..

[15] Reymbaut A, Mezzani P, Almeida Martins João P, Topgaard D (2020). Accuracy and precision of statistical descriptors obtained from multidimensional diffusion signal inversion algorithms. NMR Biomed..

[16] Harald W, Olle S, Daniel T (2010). Self-diffusion in polymer systems studied by magnetic field-gradient spin-echo NMR methods. Prog. Nucl. Magn. Reson. Spectrosc..

[17] Cory, D. G., Garroway Allen, N. & Miller Joel, B. Applications of spin transport as a probe of local geometry. In: 149–150 (1990).

[18] Cheng Y, DavidG C (1999). Multiple scattering by NMR. J. Am. Chem. Soc..

[19] Komlosh ME, Horkay F, Freidlin RZ, Nevo U, Assaf Y, Basser Peter J (2007). Detection of microscopic anisotropy in gray matter and in a novel tissue phantom using double Pulsed Gradient Spin Echo MR. J. Magn. Reson..

[20] Komlosh ME, Lizak MJ, Horkay F, Freidlin RZ, Basser PJ (2008). Observation of microscopic diffusion anisotropy in the spinal cord using double-pulsed gradient spin echo MRI. Magn. Reson. Med..

[21] Koch Martin A, Jürgen F (2008). Compartment size estimation with double wave vector diffusion-weighted imaging. Magn. Reson. Med..

[22] Daniel T (2019). Diffusion tensor distribution imaging. NMR Biomed..

[23] Daniel T (2017). Multidimensional diffusion MRI. J. Magn. Reson..

[24] De Almeida Martins João P, Topgaard D (2016). Two-dimensional correlation of isotropic and directional diffusion using NMR. Phys. Rev. Lett..

[25] Reymbaut, A., Valcourt, C. A. & Gilbert, G. *et al*. Magic DIAMOND: Multi-Fascicle Diffusion Compartment Imaging with Tensor Distribution Modeling and Tensor-Valued Diffusion Encoding (2020). 10.1016/j.media.2021.101988 33611054

[26] Reymbaut, A. Matrix moments of the diffusion tensor distribution (2020).

[27] Nørhøj JS (2012). Equivalence of double and single wave vector diffusion contrast at low diffusion weighting. NMR Biomed..

[28] Jespersen Sune N, Lundell H, Sønderby Casper K, Dyrby Tim B (2013). Orientationally invariant metrics of apparent compartment eccentricity from double pulsed field gradient diffusion experiments. NMR Biomed..

[29] Carl-Fredrik W, Hans K, Ofer P (2016). Q-space trajectory imaging for multidimensional diffusion MRI of the human brain. NeuroImage.

[30] Basser PJ, Pajevic S (2003). A normal distribution for tensor-valued random variables: Applications to diffusion tensor MRI. IEEE Trans. Med. Imaging.

[31] Freidlin Raisa Z, Özarslan E, Komlosh Michal E (2007). Parsimonious model selection for tissue segmentation and classification applications: A study using simulated and experimental DTI data. IEEE Trans. Med. Imaging.

[32] Basser Peter J, Sinisa P (2007). Spectral decomposition of a 4th-order covariance tensor: Applications to diffusion tensor MRI. Signal Process..

[33] Avram AV, Sarlls JE, Basser PJ (2019). Measuring non-parametric distributions of intravoxel mean diffusivities using a clinical MRI scanner. NeuroImage.

[34] Jaynes ET (1957). Information theory and statistical mechanics. Phys. Rev..

[35] Bai R, Cloninger A, Czaja W, PeterJ B (2015). Efficient 2D MRI relaxometry using compressed sensing. J. Magn. Reson..

[36] Furuyama Jon K, Wilson Neil E, Burns Brian L, Nagarajan R, Margolis Daniel J, Thomas MA (2012). Application of compressed sensing to multidimensional spectroscopic imaging in human prostate. Magn. Reson. Med..

[37] Paulsen Jeffrey L, Cho H, Cho G, Song YQ (2011). Acceleration of multi-dimensional propagator measurements with compressed sensing. J. Magn. Reson..

[38] Burnham KP, Anderson DR (1998). Model Selection and Inference : A Practical Information-Theoretic Approach.

[39] Nye JF (1985). Physical Properties of Crystals: Their Representation by Tensors and Matrices.

[40] Powell MJD (1998). Direct search algorithms for optimization calculations. Acta Numer..

[41] Virtanen P, Gommers R, Oliphant Travis E (2020). SciPy 1.0: Fundamental algorithms for scientific computing in Python. Nat. Methods.

[42] Kass RE, Raftery AE (1995). Bayes factors. J. Am. Stat. Assoc..

[43] Klaus H, Klaus H, Sven T (2015). Foundations of anisotropy for exploration seismics. Handbook of Geophysical Exploration. Section I. Seismic Exploration: Volume 22.

[44] Aganj, I., Lenglet, C. & Guillermo, S. ODF reconstruction in Q-ball imaging with solid angle consideration. In: 1398–1401 (2009). 10.1109/ISBI.2009.5193327 PMC4360965 25789084

[45] Samo L, Filip S, Stefanie E, Markus N, Daniel T (2014). Microanisotropy imaging: Quantification of microscopic diffusion anisotropy and orientational order parameter by diffusion MRI with magic-angle spinning of the q-vector. Front. Phys..

[46] Anderson TW (1962). An Introduction to Multivariate Statistical Analysis.

[47] Basser Peter J, Sinisa P (2010). Dealing with uncertainty in diffusion tensor MR data. Isr. J. Chem..

[48] Qu M, Jiang D, Lu Lucy X (2016). An optimal scheme for numerical evaluation of Eshelby tensors and its implementation in a MATLAB package for simulating the motion of viscous ellipsoids in slow flows. Comput. Geosci..

[49] Basser Peter J, Sinisa P (2000). Statistical artifacts in diffusion tensor MRI (DT-MRI) caused by background noise. Magn. Reson. Med..

[50] Daniel T, Olle S (2002). Self-diffusion in two- and three-dimensional powders of anisotropic domains: An NMR study of the diffusion of water in cellulose and starch. J. Phys. Chem. B.

[51] Does MD, Parsons EC, Gore JC (2003). Oscillating gradient measurements of water diffusion in normal and globally ischemic rat brain. Magn. Reson. Med..

[52] Fieremans E, Burcaw Lauren M, Lee Hong H, Lemberskiy G, Veraart J, Novikov Dmitry S (2016). In vivo observation and biophysical interpretation of time-dependent diffusion in human white matter. NeuroImage.

[53] Lee Hong H, Fieremans E, DmitryS N (2018). What dominates the time dependence of diffusion transverse to axons: Intra- or extra-axonal water?. NeuroImage.

[54] Marco P, Clémence L, Chloé N (2016). New paradigm to assess brain cell morphology by diffusion-weighted MR spectroscopy in vivo. Proc. Natl. Acad. Sci. U.S.A..

[55] De Swiet Thomas M, Mitra PP (1996). Possible systematic errors in single-shot measurements of the trace of the diffusion tensor. J. Magn. Reson. Ser. B.

[56] Jespersen Sune N, Olesen J, Andrada IL, Shemesh N (2019). Effects of nongaussian diffusion on “isotropic diffusion” measurements: An ex-vivo microimaging and simulation study. J. Magn. Reson..

[57] Clark Chris A, Hedehus M, Moseley Michael E (2001). Diffusion time dependence of the apparent diffusion tensor in healthy human brain and white matter disease. Magn. Reson. Med..

[58] Le Bihan D, Turner R, Douek P (1993). Is water diffusion restricted in human brain white matter? An echo-planar NMR imaging study. Neuroreport.

[59] Jones DK, Horsfield MA, Simmons A (1999). Optimal strategies for measuring diffusion in anisotropic systems by magnetic resonance imaging. Magn. Reson. Med..

[60] Skare S, Hedehus M, Moseley Michael E, Li T-Q (2000). Condition number as a measure of noise performance of diffusion tensor data acquisition schemes with MRI. J. Magn. Reson..

[61] Markus N, Filip S, Jan B (2020). Tensor-valued diffusion MRI in under 3 minutes: An initial survey of microscopic anisotropy and tissue heterogeneity in intracranial tumors. Magn. Reson. Med..

[62] Emmanuel C, Justin R (2007). Sparsity and incoherence in compressive sampling. Inverse Probl..

[63] Rajkowska G, Selemon Lynn D, Goldman-Rakic Patricia S (1998). Neuronal and glial somal size in the prefrontal cortex: A postmortem morphometric study of schizophrenia and huntington disease. Arch. Gen. Psychiatry.

[64] Ariane S, Blanche P, Tanguy D, Nikola S, Serge R, Julien C-A (2017). Axon and myelin morphology in animal and human spinal cord. Front. Neuroanat..

[65] Ong HH, Wehrli FW (2010). Quantifying axon diameter and intra-cellular volume fraction in excised mouse spinal cord with q-space imaging. NeuroImage.

[66] Harald B (1992). Group conduction velocities and nerve fibre diameters of alpha and gamma-motoneurons from lower sacral nerve roots of the dog and humans. Gen. Physiol. Biophys..

[67] Wolfram Research Inc. *Mathematica. * (2020).

[68] Hunter JD (2007). Matplotlib: A 2D graphics environment. Comput. Sci. Eng..

