# Supplementary material for: A new framework for MR diffusion tensor distribution
Source: Sci Rep. 2021 Feb 2;11:2766. doi: 10.1038/s41598-021-81264-x (PMC7854653; doi:10.1038/s41598-021-81264-x)
Supplement: Supplementary file 1 — Supplementary material 1 [file 41598_2021_81264_MOESM1_ESM.pdf]

Supplementary Material - A New Framework for MR Diffusion Tensor Distribution

Kulam Najmudeen Magdoom[1], Sinisa Pajevic[1], Gasbarra Dario[2], and Peter J. Basser[1]

[1]*Eunice Kennedy Shriver* National Institute of Child Health and Human Development, National Institutes of Health, Bethesda, MD, USA

[2]University of Helsinki, Finland

1 Maximum entropy property and identifiability of the CNTVD

Theorem 1. *For a Borel-measurable set, $\mathcal{C} \subseteq \mathbb{R}^d$, the constrained Gaussian distribution with density,*

$$p_{\mu,\Omega}(X|X \in \mathcal{C}) = \frac{1}{Z(\mu,\Omega)} \exp\left(-\frac{1}{2}(X-\mu)\Omega(X-\mu)^\top\right) \mathbf{1}(X \in \mathcal{C})$$

where $Z(\mu,\Omega)$ is the normalizing constant is the unique maximizer of the entropy

$$H(p) = -\int_{\mathcal{C}} p(X) \log p(X) dX$$

among all probability densities supported by $\mathcal{C}$ with given first and second moments

$$\langle X_k \rangle = \mathbb{E}_{\mu,\Omega}(X_k|X \in \mathcal{C}), \quad \langle X_k X_\ell \rangle = \mathbb{E}_{\mu,\Omega}(X_k X_\ell|X \in \mathcal{C}), \quad 1 \leq k, \ell \leq d.$$

When $\mathcal{C}$ contains an open ball the parameters μ, Ω are uniquely determined by the first and second moments.

Proof. Consider the following Lagrangian with the constraint $p(X) \geq 0$,

$$\mathcal{L}(p,\lambda,\nu,\Xi) = -\int_{\mathcal{C}} p(X) \log p(X) dX + \left(1 - \int_{\mathcal{C}} p(X) dX\right)\lambda + \left(\langle X \rangle - \int_{\mathcal{C}} X p(X) dX\right) \cdot \nu \tag{1}$$

$$+ \operatorname{Trace}\left[\left(\langle X^\top X \rangle - \int_{\mathcal{C}} X^\top X p(X) dX\right)\Xi\right] \tag{2}$$

where $\lambda \in \mathbb{R}, \nu \in \mathbb{R}^d$ and $\Xi \in \mathbb{R}^{d \times d}_{symm}$ are the Lagrange multipliers. A solution of the maximization

problem corresponds necessarily to a zero of the variational derivatives,

$$\frac{\delta \mathcal{L}}{\delta p(X)} = -\log p(X) - 1 + \lambda + X \cdot \nu + X \Xi X^\top = 0 \quad \forall X \in \mathcal{C} \tag{3}$$

which means that when the maximum entropy solution exists necessarily it has the form

$$p(X) = \exp\Big(\lambda - 1 + X \cdot \nu + X \Xi X^\top\Big) \mathbf{1}(X \in \mathcal{C}) \tag{4}$$

with λ corresponding to a normalizing constant and ν, Ξ such that the first and second moment constraints are satisfied. When a solution with the given moments exists, after reparametrization, the density is rewritten as

$$p(X) = \frac{1}{Z(\mu, \Omega)} \exp\Big(-\frac{1}{2}(X - \mu)\Omega(X - \mu)^\top\Big) \mathbf{1}(X \in \mathcal{C}). \tag{5}$$

When the support $\mathcal{C}$ is unbounded, necessarily Ω is positive definite on the recession cone of $\mathcal{C}$, and when Ω is positive definite on $\mathbb{R}^d$, we obtain a constrained Gaussian distribution. When a maximum exists, the uniqueness of the maximizing parameters is shown below.

Suppose that $p_{\widetilde{\mu}, \widetilde{\Omega}}(X)$ is another constrained Gaussian distribution with support $\mathcal{C}$ satisfying the moment constraints and maximizing the entropy. Then since the distributions satisfy the same moment constraints,

$$\nabla \log Z(\mu, \Omega) = \nabla \log Z(\widetilde{\mu}, \widetilde{\Omega}) = \big(\langle X \rangle, \langle X^\top X \rangle\big) \tag{6}$$

which implies

$$\begin{aligned} 0 &= \big(\nabla \log Z(\mu, \Omega) - \nabla \log Z(\widetilde{\mu}, \widetilde{\Omega})\big)\big(\mu - \widetilde{\mu}, \Omega - \widetilde{\Omega}\big)^\top && (7) \\ &= \big(\mu - \widetilde{\mu}, \Omega - \widetilde{\Omega}\big)\ \nabla^\top \nabla \log Z(\hat{\mu}, \hat{\Omega})\ \big(\mu - \widetilde{\mu}, \Omega - \widetilde{\Omega}\big)^\top && (8) \end{aligned}$$

with $\hat{\mu} = \alpha\mu + (1 - \alpha)\widetilde{\mu}$ and $\hat{\Omega} = \alpha\Omega + (1 - \alpha)\widetilde{\Omega}$ for some $0 \le \alpha \le 1$, and

$$\begin{aligned} \nabla^\top \nabla \log Z(\hat{\mu}, \hat{\Omega}) &= \frac{\nabla^\top \nabla Z(\hat{\mu}, \hat{\Omega})}{Z(\hat{\mu}, \hat{\Omega})} - \frac{\nabla^\top Z(\hat{\mu}, \hat{\Omega}) \nabla Z(\hat{\mu}, \hat{\Omega})}{Z(\hat{\mu}, \hat{\Omega})^2} && (9) \\ &= \begin{bmatrix} \mathrm{Cov}_{\hat{\mu}, \hat{\Omega}}(X) & \mathrm{Cov}_{\hat{\mu}, \hat{\Omega}}(X^\top X, X) \\ \mathrm{Cov}_{\hat{\mu}, \hat{\Omega}}(X, X^\top X) & \mathrm{Cov}_{\hat{\mu}, \hat{\Omega}}(X^\top X, X^\top X) \end{bmatrix} && (10) \end{aligned}$$

which means that

$$\mathrm{Variance}_{\hat{\Omega}, \hat{\theta}}\Big((\mu - \widetilde{\mu})X^\top + X(\Omega - \widetilde{\Omega})X^\top\Big) = 0. \tag{11}$$

Since $p_{\hat{\mu},\hat{\Omega}}(X) > 0$ on $\mathcal{C}$,

$$\mathcal{C} \subseteq \left\{X \in \mathbb{R}^d : (\mu - \widetilde{\mu})X^\top + X(\Omega - \widetilde{\Omega})X^\top = r\right\} \tag{12}$$

for some $r \in \mathbb{R}$, which is a variety of dimension, $(d-1)$, when $\mu \neq \widetilde{\mu}$ or $\Omega \neq \widetilde{\Omega}$, contradicting the assumption of $\mathcal{C}$ having full dimension □

2 Relations between the diffusion tensor cumulants and the displacement cumulants

Let $X \in \mathbb{R}^d$ be a conditionally Gaussian displacement vector, with zero conditional mean and conditional covariance $2D$, while D is a random symmetric positive definite matrix (diffusion tensor) with probability density $p(D)$. The Characteristic Function (CF) of the Gaussian conditional law of X given D is given by

$$\mathbb{E}\big(\exp(iq \cdot X)\big|D\big) = \exp\big(-qDq^\top\big) \tag{13}$$

while the unconditional CF is obtained by integrating w.r.t. $p(D)$ the conditional CF

$$\mathbb{E}\big(\exp(iq \cdot X)\big) = \int_{\mathcal{M}_+} \exp\big(-qDq^\top\big)p(D)dD = \mathbb{E}\big(\exp(-\mathrm{Trace}(QD))\big), \quad q \in \mathbb{R}^d, \tag{14}$$

and it coincides with the Laplace Transform (LT) of the diffusion tensor distribution $p(D)$ at $Q = q^\top q$.

We introduce the following notations: the order of a multi-index $\alpha = (\alpha_1, \ldots, \alpha_d) \in \mathbb{N}^d$ is denoted by

$$|\alpha| = \sum_{j=1}^{d} \alpha_j \tag{15}$$

and $q^\alpha = \prod_{j=1}^{d} q_j^{\alpha_j}$ for $q \in \mathbb{R}^d$. The multivariate cumulants of $X = (X_1, \ldots, X_d)$ are denoted by

$$\kappa_\alpha(X) = \kappa_{|\alpha|}\big(\underbrace{X_1, \ldots, X_1}_{\alpha_1 \text{ copies}}, \underbrace{X_2, \ldots, X_2}_{\alpha_2 \text{ copies}}, \ldots, \underbrace{X_d, \ldots, X_d}_{\alpha_d \text{ copies}}\big), \tag{16}$$

so that $\kappa_{e_j+e_\ell}(X) = \kappa_2(X_j, X_\ell)$, $\kappa_{e_j+e_k+e_\ell+e_r}(X) = \kappa_4(X_j, X_k, X_\ell, X_r)$, where $\{e_1, \ldots, e_d\}$ is the cartesian basis.

The cumulant expansion of the CF of X is given by

$$\log \mathbb{E}\big(\exp(iq \cdot X)\big) = \sum_{\alpha:|\alpha|\in 2\mathbb{N}\setminus\{0\}} \frac{\kappa_\alpha(X)}{\alpha!}(-1)^{|\alpha|/2} q^\alpha \tag{17}$$

$$= \sum_{n=1}^{\infty} \frac{(-1)^n}{(2n)!} \sum_{j_1 \ldots j_{2n}=1}^{d} \kappa_{2n}(X_{j_1}, \ldots, X_{j_{2n}}) q_{j_1} \ldots q_{j_{2n}} \tag{18}$$

where all the odd moments and cumulants vanish since the law of X is reflection symmetric, while the cumulant expansion of the LT of D is given by

$$\log \mathbb{E}\big(\exp(-\text{Trace}(QD))\big) = \sum_{\beta:|\beta|>0} \frac{\kappa_\beta(D)}{\beta!}(-1)^{|\beta|} 2^{|\bar{\beta}|} Q^\beta \tag{19}$$

$$= \sum_{n=1}^{\infty} \frac{(-1)^n}{n!} \sum_{j_1 \ldots j_{2n}=1}^{d} \kappa_n(D_{j_1 j_2}, \ldots, D_{j_{2n-1} j_{2n}}) Q_{j_1 j_2} \ldots Q_{j_{2n-1} j_{2n}} \tag{20}$$

where $\beta = (\beta_{ij} : 1 \leq i \leq j \leq d) \in \mathbb{N}^{(d+1)d/2}$, and $\bar{\beta}$ is the restriction of the multi-index β to the off-diagonal entries. By comparing these expansions for rank-1 $Q = q^\top q$, we see that

$$\langle X \rangle = 0, \quad \langle X^\top X \rangle = \text{Cov}(X) = 2\langle D \rangle, \tag{21}$$

$$\kappa_4(X_i, X_j, X_k, X_\ell) = 4\big\{\text{Cov}(D_{ij}, D_{k\ell}) + \text{Cov}(D_{ik}, D_{j\ell}) + \text{Cov}(D_{i\ell}, D_{jk})\big\} \tag{22}$$

$$\kappa_{2n}(X_{j_1}, \ldots, X_{j_{2n}}) = 2^n \sum_{\pi=\{\pi_1,\ldots,\pi_n\}} \kappa_n(D_{\pi_1}, \ldots, D_{\pi_n}) \tag{23}$$

where the sum in Equation (23) is over the pairings π of $\{j_1, \ldots, j_{2n}\}$ into pairs $\pi_1, \ldots, \pi_n$, in accordance with the law of total cumulance. The even cumulants of the displacement $\kappa_\alpha(X)$ with $|\alpha| = 2n$ are, up to rescaling, symmetrizations of corresponding cumulants of the diffusion tensor $\kappa_\beta(D)$ with $|\beta| = n$.

By truncating the cumulant expansion Equation (20) of the LT of $p(D)$ up to second order terms, we obtain the LT of the NTVD with mean $\langle D \rangle$ and Covariance$(D_{ij}, D_{k\ell}) = \mathsf{C}_{ijk\ell}$, which can be used to model the signal decay in a multiple pulsed gradient experiment:

$$\frac{S(\mathsf{Q})}{S(0)} = \mathbb{E}\big[\exp\big(-\text{Trace}(\mathsf{Q}\mathsf{D})\big)\big] = \exp\left(-\text{Trace}(\mathsf{Q}\langle D \rangle) + \frac{1}{2}\sum_{ijk\ell} \mathsf{C}_{ijk\ell} \mathsf{Q}_{ij} \mathsf{Q}_{k\ell}\right) \tag{24}$$

For single pulsed gradient experiments $\mathsf{Q} = q^\top q$ has rank 1, and Equation (24) coincides with the signal decay in Diffusion Kurtosis Imaging (DKI) [1], obtained by truncating the cumulant expansion of the CF of X (Equation (17)) up to 4th order terms,

$$\frac{S(q)}{S(0)} = \exp\left(-q\overline{\mathsf{D}}q^\top + \frac{1}{6}\text{Trace}(\overline{\mathsf{D}})^2 \sum_{ijk\ell} \mathsf{K}_{ijk\ell} q_i q_j q_k q_\ell\right), \tag{25}$$

where

$$\overline{\mathsf{D}} = \langle \mathsf{D} \rangle \quad \text{and } \mathsf{K}_{ijk\ell} = \frac{\mathsf{C}_{ijk\ell} + \mathsf{C}_{ikj\ell} + \mathsf{C}_{i\ell jk}}{\mathrm{Trace}(\langle \mathsf{D} \rangle)^2}. \tag{26}$$

are respectively the diffusion tensor and the diffusion kurtosis tensor. Thus the NTVD signal model extends the DKI signal model from single to multiple pulsed experiments, and up to rescaling, the diffusion kurtosis tensor is the symmetrization of the partially symmetric NTVD covariance tensor. Both NTVD and DKI signal models are "non-physical" and break down at high b-value, since the NTVD supports also tensors which are not positive definite, and the DKI signal decay is not the CF of a probability distribution.

3 Sufficiency of rank-1 and 2 b-matrices for diffusion tensor covariance estimation

Lemma 1. *The linear space $\mathcal{T}$ of partially symmetric 4th-order d-dimensional tensors with symmetries*

$$\mathsf{T}_{jik\ell} = \mathsf{T}_{ijk\ell} = \mathsf{T}_{k\ell ij} \quad \forall 1 \le i, j, k, \ell \le d$$

is spanned by tensors of the form

$$(u \otimes u + v \otimes v) \otimes (u \otimes u + v \otimes v) \quad \textit{with } u, v \in \mathbb{R}^d,$$

where $\otimes$ denotes outer product, and by symmetrizing it follows that the tensors $u \otimes u \otimes u \otimes u$ with $u \in \mathbb{R}^d$ span the subspace of totally symmetric 4th-order tensors.

Proof. By the spectral decomposition, every $\mathsf{T} \in \mathcal{T}$ is a linear combination of at most $\frac{(d+1)d}{2}$ 4th-order tensors of the form

$$\left(\sum_{k=1}^{d} u_k \otimes u_k \right) \otimes \left(\sum_{\ell=1}^{d} u_\ell \otimes u_\ell \right) = \sum_{k,\ell=1}^{d} u_k \otimes u_k \otimes u_\ell \otimes u_\ell \tag{27}$$

$$= \frac{1}{2} \sum_{k,\ell=1}^{d} (u_k \otimes u_k + u_\ell \otimes u_\ell) \otimes (u_k \otimes u_k + u_\ell \otimes u_\ell) - d \sum_{k=1}^{d} u_k \otimes u_k \otimes u_k \otimes u_k \tag{28}$$

with $u_k \in \mathbb{R}^d$, where

$$u \otimes u \otimes v \otimes v + v \otimes v \otimes u \otimes u = (u \otimes u + v \otimes v) \otimes (u \otimes u + v \otimes v) - u \otimes u \otimes u \otimes u - v \otimes v \otimes v \otimes v \tag{29}$$

□

It can be observed that Equation (28) is a sum of outer squares of rank-1 and rank-2 b-matrices.

Thus, rank-3 b-matrices are not necessary and rank-1 b-matrices are not enough to estimate the covariance of the diffusion tensor in a multiple pulsed gradient experiment.

4 Well-posedness of signal inversion

The CNTVD is the probability distribution with given mean, $\langle \mathsf{D} \rangle$, and covariance, $\langle \mathsf{D}, \mathsf{D} \rangle$, which is maximizing the entropy (relative to the volume measure) and therefore it is determined by its first and second moments. It is also shown in Theorem 1 that the mean and covariance determine uniquely the parameters μ, Σ of the CNTVD.

In Section 3 of the supplementary material we showed diffusion tensor covariance as fourth order partially symmetric tensors spanned by tensors, $\mathsf{b} \otimes \mathsf{b}$, where b matrices are of rank 1 and 2. Below we show that from acquisitions with b-matrices of rank 1 and 2 we can unambiguously reconstruct the first and second moments of any diffusion tensor distribution, CNTVD included.

Consider the log-signal, $\log S(\mathsf{b}) = \log\langle \exp\big(-\mathrm{Trace}(\mathsf{bD})\big)\rangle$ with a rank-1 b-matrix, $\varepsilon u^\top u$, where u is an unit vector and take its derivative at $\varepsilon = 0$ using the cumulant expansion (Equation (20),

$$\partial_\varepsilon \log S(\varepsilon u^\top u)\Big|_{\varepsilon=0} = \mathrm{Trace}(\langle \mathsf{D} \rangle u^\top u) = u \langle \mathsf{D} \rangle u^\top \tag{30}$$

Since (by spectral theorem) rank-1 matrices span the symmetric matrices, it follows that rank-1 b-matrices and zero b-value are enough to determine the mean tensor and S_0.

Consider the log-signal, $\log S(\varepsilon \mathsf{b})$, with scalar $\varepsilon > 0$, and non-negative matrix b, and take its second derivatives at $\varepsilon = 0$ using the cumulant expansion (Equation (20)),

$$\partial^2_{\varepsilon\varepsilon} \log S(\varepsilon \mathsf{b})\Big|_{\varepsilon=0} = \lim_{\varepsilon \downarrow 0} \frac{\log S(2\varepsilon \mathsf{b}) + \log S(0) - 2 \log S(\varepsilon \mathsf{b})}{\varepsilon^2} = \mathsf{b} : \langle \mathsf{D}, \mathsf{D} \rangle : \mathsf{b} \tag{31}$$

Now because in Section 3 Lemma 1 the outer products $\mathsf{b} \otimes \mathsf{b}$ with b of rank-1 and rank-2 span the space of fourth order covariance tensors, in principle data from rank-1 and rank-2 b-matrices are sufficient to estimate the covariance of DTD. Since the CNTVD is fully described by the mean and covariance tensors, the above proof shows that the signal inversion is well-posed using rank-1 and rank-2 b-matrices.

5 Model selection hierarchy

The diffusion weighted data is fitted with the following family of nested models for the mean [2] tensor and covariance tensor [3] in the decreasing order of model parsimony as shown below. The number of parameters including S_0 along with the structures of mean and covariance tensors for each model symmetry is listed in the table below.

Number of parameters	Symmetry (Mean tensor)	Symmetry (Covariance tensor)	Structure of the mean tensor	Structure of the covariance tensor in Voigt form
1	None	None	$\begin{pmatrix} 0 & 0 & 0 \\ 0 & 0 & 0 \\ 0 & 0 & 0 \end{pmatrix}$	$\begin{pmatrix} 0 & 0 & 0 & 0 & 0 & 0 \\ 0 & 0 & 0 & 0 & 0 & 0 \\ 0 & 0 & 0 & 0 & 0 & 0 \\ 0 & 0 & 0 & 0 & 0 & 0 \\ 0 & 0 & 0 & 0 & 0 & 0 \\ 0 & 0 & 0 & 0 & 0 & 0 \end{pmatrix}$
2	Isotropic	None	$p_1 \begin{pmatrix} 1 & 0 & 0 \\ 0 & 1 & 0 \\ 0 & 0 & 1 \end{pmatrix}$	$\begin{pmatrix} 0 & 0 & 0 & 0 & 0 & 0 \\ 0 & 0 & 0 & 0 & 0 & 0 \\ 0 & 0 & 0 & 0 & 0 & 0 \\ 0 & 0 & 0 & 0 & 0 & 0 \\ 0 & 0 & 0 & 0 & 0 & 0 \\ 0 & 0 & 0 & 0 & 0 & 0 \end{pmatrix}$
5	Transverse isotropic	None	$\alpha \hat{r}\hat{r}^\top + \beta \mathbf{I}$	$\begin{pmatrix} 0 & 0 & 0 & 0 & 0 & 0 \\ 0 & 0 & 0 & 0 & 0 & 0 \\ 0 & 0 & 0 & 0 & 0 & 0 \\ 0 & 0 & 0 & 0 & 0 & 0 \\ 0 & 0 & 0 & 0 & 0 & 0 \\ 0 & 0 & 0 & 0 & 0 & 0 \end{pmatrix}$
7	General Anisotropic	None	$\begin{pmatrix} D_{xx} & D_{xy} & D_{xz} \\ D_{xy} & D_{yy} & D_{yz} \\ D_{xz} & D_{yz} & D_{zz} \end{pmatrix}$	$\begin{pmatrix} 0 & 0 & 0 & 0 & 0 & 0 \\ 0 & 0 & 0 & 0 & 0 & 0 \\ 0 & 0 & 0 & 0 & 0 & 0 \\ 0 & 0 & 0 & 0 & 0 & 0 \\ 0 & 0 & 0 & 0 & 0 & 0 \\ 0 & 0 & 0 & 0 & 0 & 0 \end{pmatrix}$
$k_{mean} + 2$	Chosen symmetry	Isotropic	Corresponding structure	$\begin{pmatrix} p_1 & p_2 & p_2 & 0 & 0 & 0 \\ p_2 & p_1 & p_2 & 0 & 0 & 0 \\ p_2 & p_2 & p_1 & 0 & 0 & 0 \\ 0 & 0 & 0 & 2(p_1-p_2) & 0 & 0 \\ 0 & 0 & 0 & 0 & 2(p_1-p_2) & 0 \\ 0 & 0 & 0 & 0 & 0 & 2(p_1-p_2) \end{pmatrix}$

$k_{mean}+3$	Chosen symmetry	Cubic	Corresponding structure	$\begin{pmatrix} p_1 & p_2 & p_2 & 0 & 0 & 0 \\ p_2 & p_1 & p_2 & 0 & 0 & 0 \\ p_2 & p_2 & p_1 & 0 & 0 & 0 \\ 0 & 0 & 0 & p_3 & 0 & 0 \\ 0 & 0 & 0 & 0 & p_3 & 0 \\ 0 & 0 & 0 & 0 & 0 & p_3 \end{pmatrix}$
$k_{mean}+5$	Chosen symmetry	Hexagonal	Corresponding structure	$\begin{pmatrix} p_1 & p_2 & p_3 & 0 & 0 & 0 \\ p_2 & p_1 & p_3 & 0 & 0 & 0 \\ p_3 & p_3 & p_4 & 0 & 0 & 0 \\ 0 & 0 & 0 & p_5 & 0 & 0 \\ 0 & 0 & 0 & 0 & p_5 & 0 \\ 0 & 0 & 0 & 0 & 0 & 2(p_1-p_2) \end{pmatrix}$
$k_{mean}+6$	Chosen symmetry	Trigonal I	Corresponding structure	$\begin{pmatrix} p_1 & p_2 & p_3 & p_4 & 0 & 0 \\ p_2 & p_1 & p_3 & -p_4 & 0 & 0 \\ p_3 & p_3 & p_5 & 0 & 0 & 0 \\ p_4 & -p_4 & 0 & p_6 & 0 & 0 \\ 0 & 0 & 0 & 0 & p_6 & 2p_4 \\ 0 & 0 & 0 & 0 & 2p_4 & 2(p_1-p_2) \end{pmatrix}$
$k_{mean}+6$	Chosen symmetry	Tetragonal I	Corresponding structure	$\begin{pmatrix} p_1 & p_2 & p_3 & 0 & 0 & 0 \\ p_2 & p_1 & p_3 & 0 & 0 & 0 \\ p_3 & p_3 & p_4 & 0 & 0 & 0 \\ 0 & 0 & 0 & p_5 & 0 & 0 \\ 0 & 0 & 0 & 0 & p_5 & 0 \\ 0 & 0 & 0 & 0 & 0 & p_6 \end{pmatrix}$
$k_{mean}+7$	Chosen symmetry	Trigonal II	Corresponding structure	$\begin{pmatrix} p_1 & p_2 & p_3 & p_4 & -p_5 & 0 \\ p_2 & p_1 & p_3 & -p_4 & p_5 & 0 \\ p_3 & p_3 & p_6 & 0 & 0 & 0 \\ p_4 & -p_4 & 0 & p_7 & 0 & 2p_5 \\ -p_5 & p_5 & 0 & 0 & p_7 & 2p_4 \\ 0 & 0 & 0 & 2p_5 & 2p_4 & 2(p_1-p_2) \end{pmatrix}$
$k_{mean}+7$	Chosen symmetry	Tetragonal II	Corresponding structure	$\begin{pmatrix} p_1 & p_2 & p_3 & 0 & 0 & p_4 \\ p_2 & p_1 & p_3 & 0 & 0 & -p_4 \\ p_3 & p_3 & p_5 & 0 & 0 & 0 \\ 0 & 0 & 0 & p_6 & 0 & 0 \\ 0 & 0 & 0 & 0 & p_6 & 0 \\ p_4 & -p_4 & 0 & 0 & 0 & p_7 \end{pmatrix}$

$k_{mean} + 9$	Chosen symmetry	Orthorhombic	Corresponding structure	$\begin{pmatrix} p_1 & p_2 & p_3 & 0 & 0 & 0 \\ p_2 & p_4 & p_5 & 0 & 0 & 0 \\ p_3 & p_5 & p_6 & 0 & 0 & 0 \\ 0 & 0 & 0 & p_7 & 0 & 0 \\ 0 & 0 & 0 & 0 & p_8 & 0 \\ 0 & 0 & 0 & 0 & 0 & p_9 \end{pmatrix}$
$k_{mean}+13$	Chosen symmetry	Monoclinic I	Corresponding structure	$\begin{pmatrix} p_1 & p_2 & p_3 & 0 & p_{10} & 0 \\ p_2 & p_4 & p_5 & 0 & p_{11} & 0 \\ p_3 & p_5 & p_6 & 0 & p_{12} & 0 \\ 0 & 0 & 0 & p_7 & 0 & p_{13} \\ p_{10} & p_{11} & p_{12} & 0 & p_8 & 0 \\ 0 & 0 & 0 & p_{13} & 0 & p_9 \end{pmatrix}$
$k_{mean}+13$	Chosen symmetry	Monoclinic II	Corresponding structure	$\begin{pmatrix} p_1 & p_2 & p_3 & 0 & 0 & p_{10} \\ p_2 & p_4 & p_5 & 0 & 0 & p_{11} \\ p_3 & p_5 & p_6 & 0 & 0 & p_{13} \\ 0 & 0 & 0 & p_7 & p_{12} & 0 \\ 0 & 0 & 0 & p_{12} & p_8 & 0 \\ p_{10} & p_{11} & p_{13} & 0 & 0 & p_9 \end{pmatrix}$
$k_{mean}+21$	Chosen symmetry	Triclinic	Corresponding structure	$\begin{pmatrix} p_1 & p_2 & p_3 & p_4 & p_5 & p_6 \\ p_2 & p_7 & p_8 & p_9 & p_{10} & p_{11} \\ p_3 & p_8 & p_{12} & p_{13} & p_{14} & p_{15} \\ p_4 & p_9 & p_{13} & p_{16} & p_{17} & p_{18} \\ p_5 & p_{10} & p_{14} & p_{17} & p_{19} & p_{20} \\ p_6 & p_{11} & p_{15} & p_{18} & p_{20} & p_{21} \end{pmatrix}$

Table 1: Hierarchy of models used to estimate the mean and covariance tensors of the DTD. The symbol p_i refers to the ith parameter, and $\alpha, \beta, \hat{r}, \mathsf{I}$ in the structure of mean tensor of transverse isotropy symmetry are constant, constant, unit radial vector defined by the polar and azimuthal angles and idendity matrix respectively, and k_{mean} refers to the number of parameters in the chosen symmetry for the mean tensor.

6 Evaluation of heterogeneity stains

The efficacy of proposed stains in describing the desired heterogeneity were evaluated by controlled mixing of diffusion tensor ellipsoids of different size, shape and orientation. Different populations of tensors were mixed in a voxel to test if changes in the proposed heterogeneity measures correlate with changes in heterogeneity of interest, and the absolute value of the metric reflects the extent of desired heterogeneity. The following six populations (N = 1000) of diffusion tensors were generated for mixing : 1) "RandomShapes" - randomly shaped diffusion tensors of same orientation and trace

equal to T_1, 2) “RandomShapes Resized” - “RandomShapes” class of diffusion tensors with trace modified to $5T_1$, 3) “RandomShapes Rotated” - “RandomShapes” class of diffusion tensors rotated by $90°$ around the axis, $[1, 1, 1]$, 4) “Sphere” - identical spherical diffusion tensors with trace equal to T_3, 5) “Oblate” - identical oblate diffusion tensors with trace equal to $T_4 = T_3$, 6) “ProlateRand” - prolate diffusion tensors with trace equal to $T_5 = T_4 - 4$ and are rotated randomly.

The size heterogeneity measure (V_{size}) is evaluated by mixing “RandomShapes” and “RandomShapes Resized” class of diffusion tensors. The shape heterogeneity measure (V_{shape}) is evaluated by mixing Oblate and Sphere class of diffusion tensors. The orientation heterogeneity measure (V_{orient}) is evaluated by mixing “RandomShapes” and “RandomShapes Rotated” class of diffusion tensors. Finally, “Oblate” and “ProlateRand” class of diffusion tensors are mixed together to evaluate the efficacy of the stains in filtering size, shape and orientation heterogeneities in the resulting mixture. The mixing fraction is gradually increased from 0 (all tensors belonging to the first group) to 1 (all tensors belonging to the second group) for controlled mixing of two groups.

The results of the mixing is shown in Figure 1 below. It can be observed that in the first case (“RandomShapes” vs “RandomShapes Resized”), all the stains except the size heterogeneity stain change with mixing fraction. The trend observed in the size heterogeneity stain was also parabolic with maximum attained at mixing fraction equal to 0.5 corresponding to the mixture with maximum entropy which reflects the ability of the stain to accurately measure the extent of size mixing of diffusion tensors. The shape heterogeneity stain was uniformly high due to the randomizing of shapes of both the mixing population. The shape heterogeneity measure in the second case (“Oblate” vs “Sphere”) also exhibited a parabolic trend with mixing fraction but zero value for size and orientation heterogeneity measures as expected. The orientation heterogeneity measure in the third case (“RandomShapes” and “RandomShapes Rotated) also exhibited a parabolic trend with mixing fraction while the shape measure was uniform and above zero due to the presence of shape heterogeneity in both the mixing populations. The fourth case clearly showed parabolic trends for size and shape heterogeneity measures and approximately linear trend for the orientation measure as expected in the fully heterogeneous mixture.

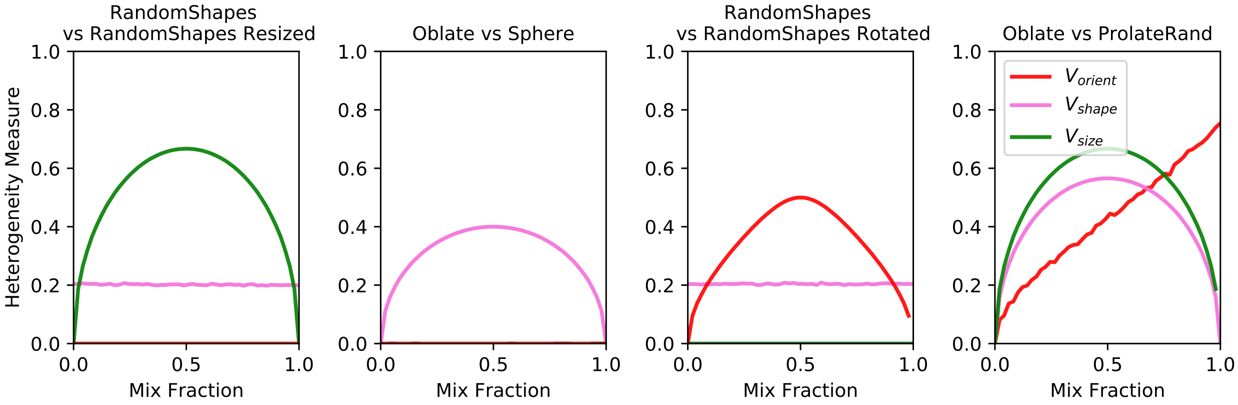

Figure 1: Efficacy of size (V_{size}), shape (V_{shape}) and orientation (V_{orient}) heterogeneity stains in independently capturing the desired heterogeneity. The plot of heterogeneity measures versus the mixing fraction computed for mixture of two groups of : (First column) randomly shaped tensors of same orientation but different sizes, (Second column) oblate and spherical tensors of same size, (Third column) randomly shaped tensors of same size but rotated by 90° around the axis, [1,1,1], and (Fourth column) oblate and randomly rotated prolates of different sizes. The above graphics was generated using the *matplotlib* package [4]

References

[1] Tabesh Ali, Jensen Jens H., Ardekani Babak A., Helpern Joseph A.. Estimation of tensors and tensor-derived measures in diffusional kurtosis imaging. *Magnetic Resonance in Medicine.* 2011;65(3):823–836.

[2] Freidlin Raisa Z., Özarslan Evren, Komlosh Michal E., et al. Parsimonious model selection for tissue segmentation and classification applications: A study using simulated and experimental DTI data. *IEEE Transactions on Medical Imaging.* 2007;26(11):1576–1584.

[3] Nye J.F. *Physical properties of crystals: their representation by tensors and matrices.* Oxford University Press; 1985.

[4] Hunter John D.. Matplotlib: A 2D graphics environment. *Computing in Science and Engineering.* 2007;9(3):99–104.
